# Possible frequent multiple mitochondrial DNA copies in a single nucleoid in HeLa cells

**DOI:** 10.1038/s41598-023-33012-6

**Published:** 2023-04-08

**Authors:** Vojtěch Pavluch, Tomáš Špaček, Hana Engstová, Andrea Dlasková, Petr Ježek

**Affiliations:** Department of Mitochondrial Physiology, No. 75, Institute of Physiology, Academy of Sciences of the Czech Republic, Prague, Czech Republic

**Keywords:** DNA, Bioenergetics, Biological fluorescence, Single-molecule biophysics, Mitochondria, Biochemistry, Molecular medicine

## Abstract

Previously, a number of ~ 1.4 of mitochondrial DNA (mtDNA) molecules in a single nucleoid was reported, which would reflect a minimum nucleoid division. We applied 3D-double-color direct stochastic optical reconstruction microscopy (dSTORM), i.e. nanoscopy with ~ 25–40 nm x,y-resolution, together with our novel method of Delaunay segmentation of 3D data to identify unbiased 3D-overlaps. Noncoding D-loops were recognized in HeLa cells by mtDNA fluorescence in situ hybridization (mtFISH) 7S-DNA 250-bp probe, containing biotin, visualized by *anti*-biotin/Cy3B-conjugated antibodies. Other mtFISH probes with biotin or Alexa Fluor 647 (A647) against *ATP6*-*COX3* gene overlaps (1,100 bp) were also used. Nucleoids were imaged by *anti*-DNA/(A647-)-Cy3B-conjugated antibodies. Resulting histograms counting mtFISH-*loci*/nucleoid overlaps demonstrated that 45% to 70% of visualized nucleoids contained two or more D-loops or *ATP6*-*COX3*-*loci,* indicating two or more mtDNA molecules *per* nucleoid. With increasing number of mtDNA *per* nucleoid, diameters were larger and their distribution histograms peaked at ~ 300 nm. A wide nucleoid diameter distribution was obtained also using 2D-STED for their imaging by *anti*-DNA/A647. At unchanged mtDNA copy number in osteosarcoma 143B cells, TFAM expression increased nucleoid spatial density 1.67-fold, indicating expansion of existing mtDNA and its redistribution into more nucleoids upon the higher TFAM/mtDNA stoichiometry. Validation of nucleoid imaging was also done with two TFAM mutants unable to bend or dimerize, respectively, which reduced both copy number and nucleoid spatial density by 80%. We conclude that frequently more than one mtDNA molecule exists within a single nucleoid in HeLa cells and that mitochondrial nucleoids do exist in a non-uniform size range.

## Introduction

Mitochondria form a highly connected network within the cell, which becomes fragmented under certain physiological states (cell division, mitophagy) and pathological conditions^1–7^. Mitochondria possess an autonomous genome, in mammals encoding ND1–ND6 and ND4L subunits of the respiratory chain complex I, CYT b of complex III, COX1–COX3 of complex IV and two membrane-embedded subunits of the ATP-synthase ATP6 and ATP8. Thus double-strand (ds) mitochondrial DNA (mtDNA) encodes for 13 proteins, 22 tRNAs and 2 rRNAs^6–15^. In this way, mitochondria exert a significant control over the cell, such as during initiation of apoptosis, metabolic switch upon hypoxia and oncogenic transformation, redox and retrograde signalling, etc., representing the metabolic and information hub. Mutations of mtDNA and impairment of mt genome expression lead to numerous diseases^15–17^.

Moreover, mtDNA is organized in the form of nucleo-protein complexes termed nucleoids^18–23^, which contain yet undefined number of copies of double-stranded (ds) mtDNA. Biology of nucleoids is still largely unknown. Likewise, not much is known about nucleoid division and segregation of mtDNA. Most probably, replication of mtDNA proceeds in nucleoids in a proximity to endoplasmic reticulum (ER), at ER-mitochondria contact sites^5^. A hypothetical division of nucleoids can be facilitated by mitochondrial fission, when mitochondrial network fragments are being moved apart^3^.

In nucleoids, mtDNA is highly packed. Partial unwinding and hence diffuse nucleoid structure allows mtDNA replication and transcription. The main binding partner of highly packed mtDNA is TFAM, a mitochondrial a transcription factor. TFAM bends mtDNA and crosslinks its chains, to allow for tight mtDNA packing, such as observed otherwise only in prokaryotes^21–25^. TFAM is also required for mtDNA transcription and replication. Both mtDNA transcription and replication are considered to proceed continuously. They oppose the processes of mitophagy and mtDNA degradation, establishing homeostasis. Other gene expression machinery and mtDNA repair proteins permanently exist within or are recruited to the nucleoid structure^18–20,23^.

Experiments showed the non-existence of mtDNA mixing between nucleoids, which can be explained by the existence of a single copy of ds mtDNA within a single nucleoid^26^. A precise number of mtDNA molecules in a single nucleoid (*n*_mtDNA_) is still a controversial issue. Originally, a ratio of six mtDNA molecules *per* nucleoid has been reported on average^18,19^. Later, this ratio has been refined to 1.4 mtDNA molecules *per* nucleoid based on 2D stimulated emission depletion microscopy (2D STED)^27–29^. STED was employed for imaging of nucleoids in typically flat cells and *n*_mtDNA_ was calculated from simultaneous evaluation of mtDNA copy number (*C*_N_), *i.e*. number of mtDNA molecules within a single cell. Theoretically, determining mtDNA copy number within a single cell with simultaneous estimation of the number of nucleoids within that particular cell would lead to precise evaluation of (an average) number of mtDNA molecules *per* nucleoid. The mtDNA copy number *C*_N_ has been also directly visualized by various microscopy techniques observing for example that *C*_N_ varies between different tissues^30^, while quite low values are typical for some cancers^31^. *C*_N_ also increases during the S-phase in the cell cycle^32^.

Despite the advances of super-resolution microscopy^27,28,33,34^ and 3D super-resolution imaging modalities^35–42^, even their application did not resolve this issue yet. As a rule, super-resolution microscopy studies based on scanning (albeit with depleting beam in the stimulation depletion, STED microscopy) typically reported images of nucleoids of approximately uniform size^27,28^, whereas the stochastic-based microscopic methods showed nucleoids of a wide distribution of sizes^33–42^, which was confirmed by FIB/SEM or cryo electron microscopy^28,35^. The discrepancy concerning *n*_mtDNA_ can also originate from the way of nucleoid imaging as such. When nucleoid proteins were artificially conjugated with protein fluorophores, one would argue that such setting of non-physiological conditions altered original nucleoid size and distribution. Also, when proteins were visualized immuno-cytochemically, the resulting nucleoid images contour the protein shell of nucleoids, the size of which and content of recruited proteins may be independent of the mtDNA content. Hence, the only way is to define the minimum nucleoid size as the spatial extent of mtDNA^38,40,41^.

Nucleoids of sizes between 50 to > 200 nm were also found by 4Pi-single-molecule switching microscopy^41^ as well as direct stochastic optical reconstruction microscopy (dSTORM,^38,40^). Even by conventional microscopy, hundreds to thousands nucleoids were found in a single cell, being distributed in the matrix, *i.e*. interior of the mitochondrial reticulum network^30,32,43,44^. Nevertheless, mtDNA replication should lead transiently to mtDNA doubling within a nucleoid, at least until the moment when nucleoid division (still elusive and unobserved) is finished^38^. As a result, any number over 1 (twice over an estimated average number, *n*_mtDNA_) should indicate an extent of mtDNA replication, when considering model of a single mtDNA *per* a single nucleoid (*n*_mtDNA_ *per* a single nucleoid, respectively).

An alternative to DNA intercalating dyes^30^ and DNA immunocytochemistry seems to be fluorescence in situ hybridization (FISH) of particular DNA sequences^45^. To improve the precision in counting mtDNA within nucleoids, we developed prototype of mtDNA and D-loop hybridization probes for mitochondrial FISH (mtFISH) and visualized them by 3D dSTORM^38–40,46^. Having acquired a double channel acquisition, yielding 3D double-color images, we thus visualized nucleoids together with the desired mtDNA *loci*. Either the D-loops or *ATP6*-*COX* sequence parts of mtDNA were visualized together with the entire mtDNA core, stained e.g. with *anti*-DNA antibodies. Using a novel procedure for 3D spatial overlaps (Supplemental Information Part I, Fig. S1–S7), we counted the number of the visualized *loci* (such as D-loops) *per* a single visualized nucleoid, which yielded *n*^*i*^_mtDNA_ for each *i*-th visualized nucleoid separately. The obtained distribution histograms for % content of *n*_mtDNA_ of 2, 3, 4, etc., relatively to all imaged nucleoids showed that a large fraction of nucleoids hybridized to two or more mtFISH probes. Validation of 3D dSTORM nucleoid imaging was done with mutant TFAM. We conclude that frequently more than one mtDNA molecule exists within a single nucleoid and that mitochondrial nucleoids do exist in a non-uniform size range.

## Results

### Design of mtDNA fluorescence in situ hybridization (mtFISH) probes for 3D double-color dSTORM imaging

**Figure 1:**
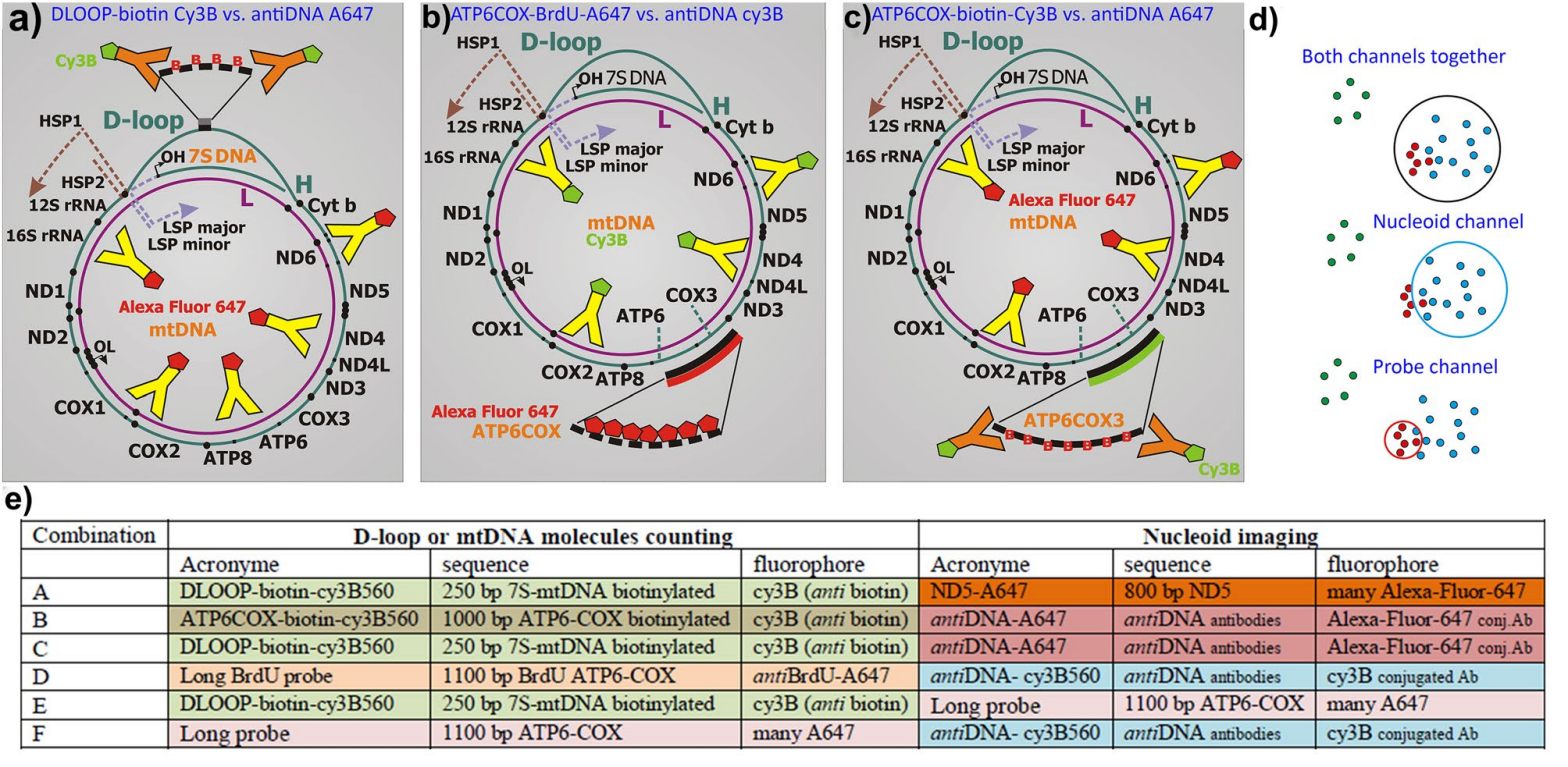
Combinations of mtFISH and immunostaining for double-color 3D dSTORM. Schemes of circular ds mtDNA are displayed, with indicated targets of the prepared hybridization probes and employed antibodies, for following combinations: (**a**) DLOOP-biotin Cy3B *vs*. antiDNA-A640, representing *anti*-biotin Cy3B-immunostained 250 bp biotin-containing mtFISH probe against 7S mtDNA, simultaneously with *anti*-DNA immunostaining (simplified by a single antibody “Y” conjugated with A647). (**b**) ATP6COX-biotin Cy3B *vs*. *anti*DNA-A647, for which a target is the mtDNA region spanning the majority of *ATP6* and *COX3* genes. The corresponding 1,100 bp mtFISH hybridization probe with the directly inserted biotin. This dark probe is then visualized by Cy3B-immunostaining. For nucleoids, *anti*-DNA A647-immunostaining is used. (**c**) ATP6COX-A647 *vs*. *anti*DNA-Cy3B combines the 1,100 bp mtFISH probe similar as in panel (**b**), but with the directly inserted A647, while *anti*-DNA Cy3B-immunostaining visualizes nucleoids. (**d**) Procedure of determination of overlaps in 3D space schematically depicted as it was 2D projection. Red and blue colors show the localized fluorophores (points) of the two respective optical channels taken together at first round of segmentation (top), thus identifying nucleoids with mtFISH loci together; whereas and displaying them individually in the second round—middle: nucleoid channel; bottom: mtFISH probe channel. The green points represent those ones, which were excluded by segmentation (tessellation) procedure. (**e**) Design of dSTORM double channel 3D imaging using mtFISH probes and antibody combination. The left part describes acronyms of mtFISH probes, their targets in mtDNA sequence and the fluorophore used. The right part shows antibodies for nucleoid imaging, acronyms, antigens and fluorophores.

We designed mtDNA fluorescence in situ hybridization (mtFISH) probes for three-dimensional (3D) direct stochastic optical reconstruction microscopy (dSTORM) to visualize the chosen *loci* of mtDNA within 3D-images of mtDNA nucleoids. Since single-fluorophore-containing probes did not provide any sufficient fluorescence for dSTORM, we constructed mtFISH probes with biotin, when the required signal was obtained by *anti*-biotin antibodies (Ab) and Cy3B-560-conjugated-Ab (Fig. 1a,e); or alternatively instead of biotin, we introduced Alexa Fluor 647 (A647; via A647-dUTP) directly into long length mtFISH probes (~ 1,000 bp) (Fig. 1b,c,e) to detect signals directly via the incorporated fluorophore (Fig. 1c). To visualize selected specific sequences of mtDNA, we constructed the 250 bp biotin probe against the 7S-DNA portion of the mtDNA D-loop (Fig. 1a) and the 1,100 bp biotin probe against the overlapping sequence of *ATP6* and *COX3* genes (Fig. 1b). Similar 1,100 bp *ATP6COX3* probes were designed with directly incorporated A647 (Fig. 1c). Figure 1 summarizes the employed set of mtDNA hybridization probes and their use in combination with Ab and chosen fluorophores. Probes of 250 or 1100 bp lengths carried fluorophores at each 40th nucleotide, thus contained 6 or 27 fluorophores, respectively, or biotin labels. When assumed to hybridize by 25–50 bp region, the 250 bp biotin probe can be extended beyond mtDNA up to 60 nm in an unlikely case with its free portion fully unwounded. In such a case, the freely extended portion would contain up to 5 fluorophores or biotin molecules. The latter may be recognized by even further distant Ab fluorophore.

**Figure 2:**
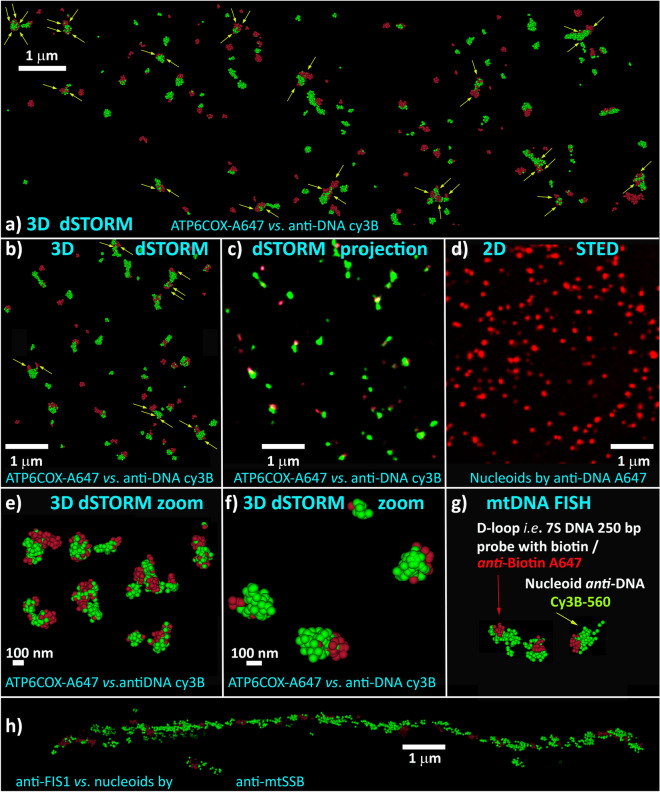
Comparison of 2D projections from double-color 3D dSTORM and 2D STED nucleoid imaging. (**a**,**b**,**c**,**e**,**f**) 3D matrix data of double color 3D dSTORM or their 2D projection in a point-splatting mode (**c**), visualizing mtDNA nucleoids using *anti*-DNA/Cy3B (*green*) and the ATP6COX 1100 bp mtFISH probe containing A647 (*red*) in a point-cloud mode (**a**–**c** and zoomed in **e**,**f**). Arrows indicate those nucleoids in which more than two overlapps with mtFISH probes exist. Exemplar nucleoids were enlarged and displayed together in panel (**e**). Panel (**g**) shows schematic of mtFISH now illustrated by “D-LOOP “ biotin-containing mtFISH 250 bp probe hybridizing to 7S DNA visualized with *anti*-Biotin/A647 (red), while nucleoids were stained using *anti*-DNA/Cy3B (*green*). (**d**) STED imaging of nucleoids via *anti*-DNA/A647 on a DMi8 inverted microscope with laser scanning confocal head Leica TCS SP8 using 660 nm continual depletion laser. Note, a thin 1000 nm z-slice for dSTORM is compared with the whole cell 2D imaging by STED, leading to apparently more nucleoids in the STED image. (**h**) Dual antibody staining for outer membrane protein FIS1 contouring mt tubules and nucleoid protein mtSSB. For methods see Ref. 38 and 40. Resolution, calculated on the basis of decorrelation analysis^53^, using the Image J plug-in^53^ was 47 nm for xy-plane of the STED image in panel (**d**); and in panel (**a**) it was 38 nm for xy-plane projection of data within the 500 nm z-axis width of the 3D dSTORM image of nucleoids (*anti*-DNA/Cy3B, *green*) as well as 38 nm for a similar projection of the data for ATP6COX 1100 bp mtFISH probe containing A647 (*red*). The complete-image data, on which a detail of panel (**g**) was based, yielded resolution of 34 nm for nucleoids and 36 nm for the 7S DNA probe visualized with *anti*-Biotin/A647 (*red*). Calculations were performed with the original 3D data not from pixels of panels (**a**,**g**).

The fluorophores were chosen based on their ability to photoblink in reducing media, hence to be suitable for 3D dSTORM nanoscopy with 25–40 nm x,y-resolution^38,40^ (Fig. S8). We used mtFISH probes in one channel (color) of the 3D dSTORM imaging acquisition, being aware of their detection via 3D-immunofluorescence. The second channel did collect fluorescence of antibodies, conjugated with a proper dye, contouring nucleoids (Fig. 2a–c,e–h). In all cases, Cy3B localizations had to be collected after those of A647. The single channel 3D data for nucleoids (including 2D projections of nucleoid models) yielded similar nucleoid wide size distribution, typically diameter distribution for spherical models, as previously described^38,40^. This also provides an inherent control that the RNAse and DNAse treatment, employed to enable FISH labelling, does not significantly distort the nucleoid distribution and does not induce nucleoid clustering. The wide size distribution was obtained even when 2D STED was used (Figs. 2d, 7f). Thus non-uniform nucleoids were imaged independently of the method. Note that in cells with minor mt network fragmentation, nucleoids are positioned in linear tubules of mt network nearly in equidistant positions of ~ 1 µm (Figs. 2h, 8g)^37,39,43^.

### More than a single copy of mtDNA may frequently exist in HeLa cell mitochondrial nucleoids

**Figure 3:**
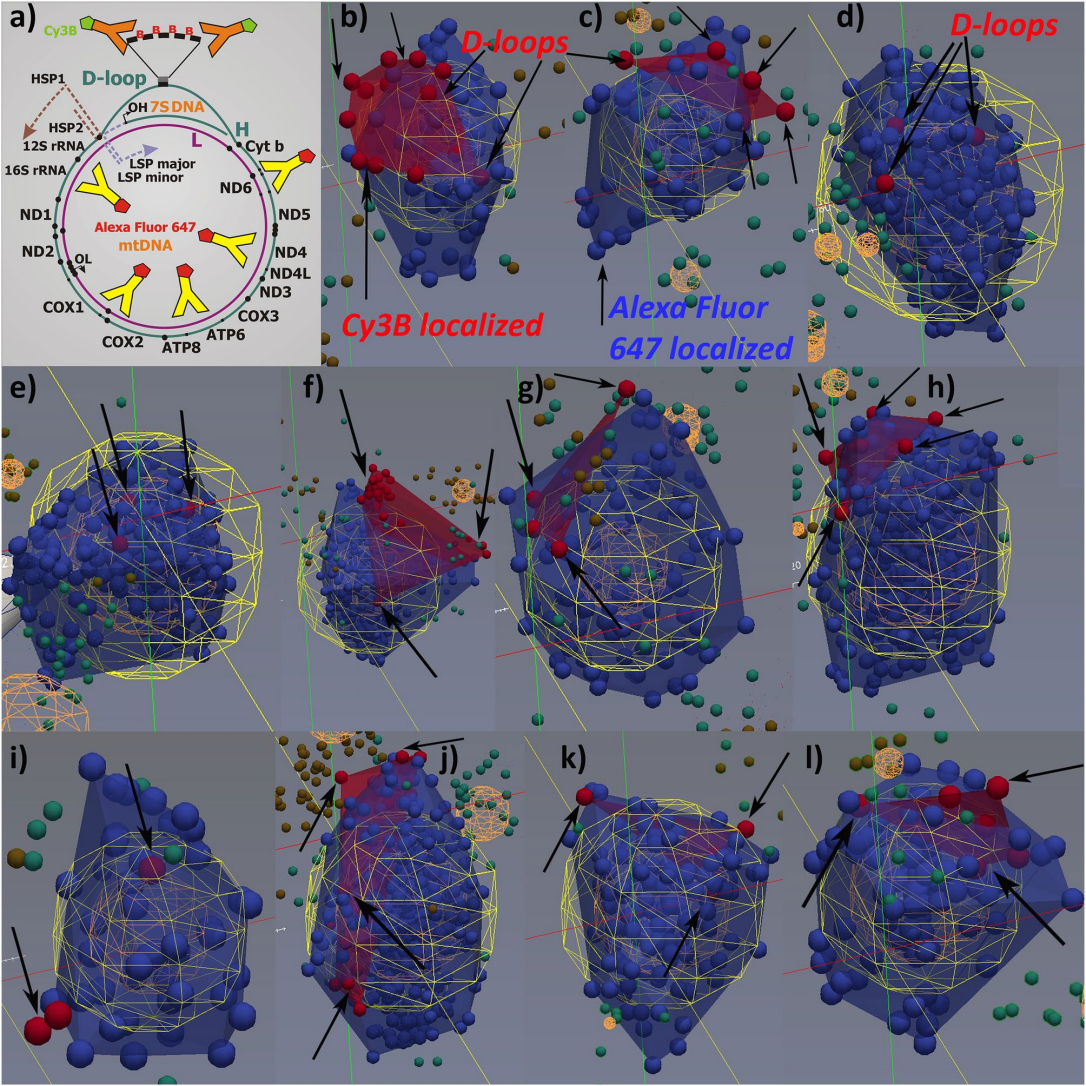
D-loops visualized together with mtDNA nucleoids as reconstructed from double 3D dSTORM–DLOOP-biotin Cy3B *vs*. antiDNA-A647 protocol. (**a**) Schematic of protocol, which is identical to that of Fig. 1a; (**b**–**l**) Reconstructed 3D images—*Blue small spheres/points* (diameter of which now represents the 20 nm scale) show localizations of Alexa-Fluor-647 after DNA immunostaining, visualizing mtDNA cores of nucleoids. These are co-localized with *red small spheres/points*, indicating localizations of Cy3B fluorophores in a proximity of D-loops. Cy3B560 was conjugated with the secondary antibodies, staining *anti*-biotin primary antibodies, while biotin was abundantly inserted into the 7S DNA hybridization probe for mtFISH. Red surface is depicted to show an extent of mtFISH regions defined by the red points. *Green* points (*orange spheres* for a group of them) in figures represent those localizations beyond the regions of segmented nucleoid and probe regions and nucleoid/probe overlaps. Delaunay parameter *A*_max_ of 60 nm was used while calculated MFD was 41.4 nm. Diameters of spheroid models of nucleoids (*yellow spheres*) were as follows (number of localized fluorophores in parentheses as well as numbers of mtFISH loci)—(**b**) 193 nm (82; 5 loci); (**c**) 169 nm (67; 5 loci); (**d**) 269 nm (246; 3 loci); (**e**) 350 nm (305; 3 loci); (**f**) 338 nm (289; 3 loci); (**g**) 227 nm (98; 3 loci); (**h**) 267 nm (401; 5 loci); (**i**) 139 nm (45; 2 loci); (**j**) 287 nm (477; > 4 loci); (**k**) spheres 25 nm, 292 nm (148; 3 loci); and (**l**) spheres 25 nm, 222 nm (93; 3 loci). Drawings were performed using ParaView software, version 4.3.1, 64 bit^51^.

**Figure 4:**
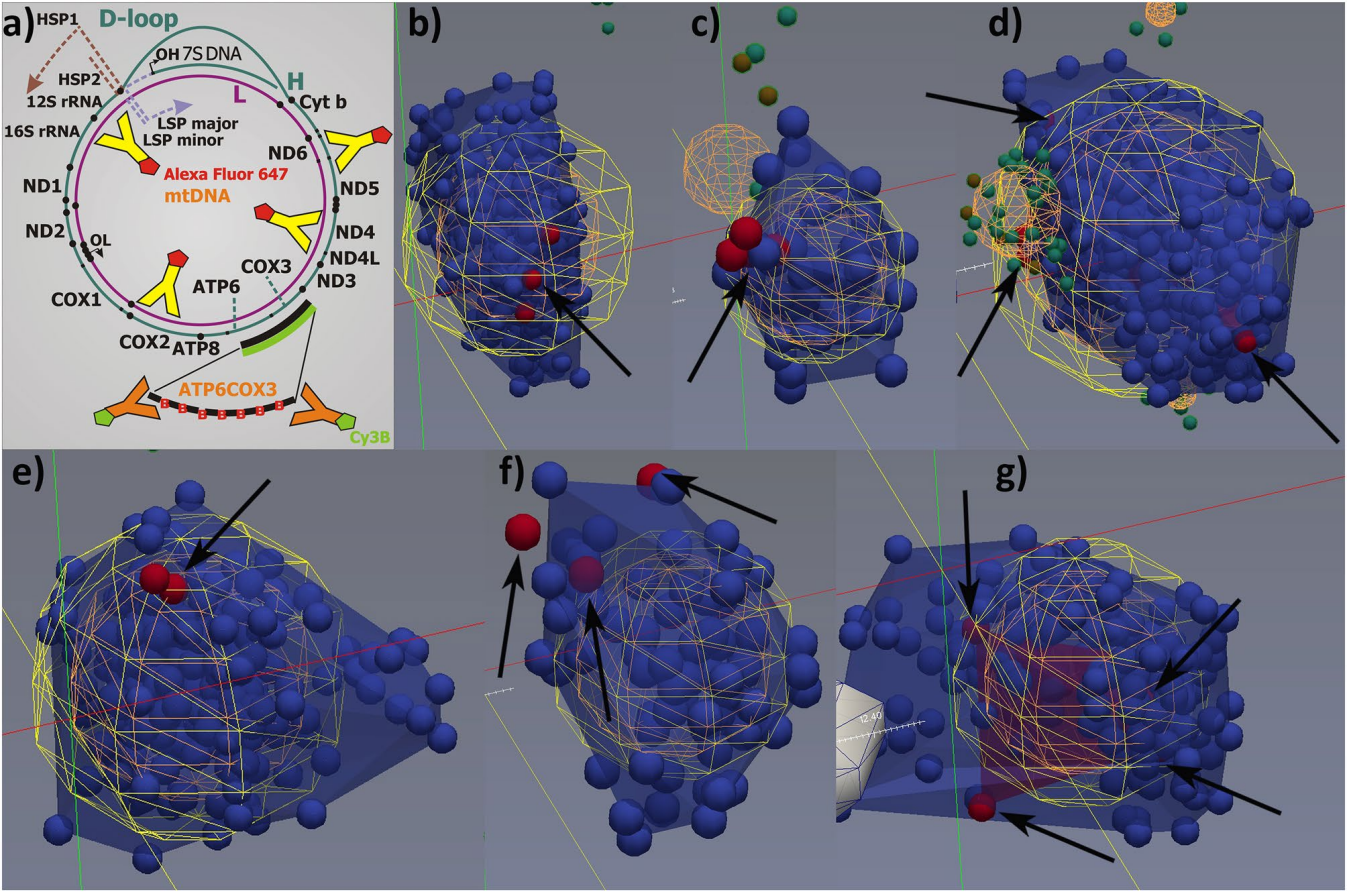
*ATP6*-*COX3* regions visualized together with mtDNA nucleoids as reconstructed from double 3D dSTORM—ATP6COX-biotin Cy3B vs. antiDNA-A647 protocol. (**a**) Scheme of protocol, which is identical to that of Fig. 1B; (**b**–**g**) Reconstructed 3D images—Color coding and object meaning as in Fig. 3: *Blue small spheres/points* (diameter of which represents the 25 nm scale) show localizations of Alexa-Fluor-647 after DNA immunostaining visualizing mtDNA spaces within nucleoids. These are co-localized with *red small spheres/points*, indicating localizations of the *ATP6*-*COX3* gene region which was target of the ATP6COX-biotin 1,100 bp hybridization probe with the directly inserted biotin, which was visualized by *anti*-biotin Cy3B-immunostaining. Thus, the second channel (*red* spheres) shows localizations of Cy3B fluorophores, *i.e*. *ATP6*-*COX3* gene regions. *Green* points (*orange spheres* for a group of them) in figures represent those localizations beyond the regions of segmented nucleoid and probe regions and the regions of nucleoid/probe overlaps. Delaunay parameter *A*_max_ of 80 nm was used while calculated MFD was 25.8 nm. Diameters of spheroid models of nucleoids (*yellow spheres*) were as follows (number of localized fluorophores in parentheses as well as numbers of mtFISH loci)—(**b**) 284 nm (481; 3 loci); (**c**) 139 nm (54; 1 locus); (**d**) 344 nm (482; 3 loci); (**e**) 261 nm (284; 1 locus); (**f**) 168 nm (78; 3 loci); and (**g**) 228 nm (204; 3 to 4 loci). Drawings were performed using ParaView software, version 4.3.1, 64 bit^51^.

Examples of double-color 3D dSTORM-visualized mitochondrial nucleoids in HeLa cells are shown together with the indicated combinations of mtDNA hybridization probes and/or antibodies in Figs. 3 and 4. Localized fluorophores are drawn as spheres of 20 and 25 nm, respectively, indicating localization uncertainty, enabling spatial resolution as low as ~ 40 nm (Fig. S8). Green points in figures represent those localizations beyond the regions of nucleoid/probe overlaps, i.e. localizations excluded by Delaunay segmentation. In Fig. 3, the 250 bp 7S-mtDNA biotin-containing hybridization probe (“DLOOP-biotin Cy3B “) was used to visualize and count D-loops. Hybridization against *ATP6-COX3 loci* was also performed, as shown in Fig. 4 (“ATP6COX-biotin Cy3B “). Thus the nucleoid images were reconstructed from ~ 100 to 400 localized fluorophore blinking (“points”), whereas fewer “points” were grouped for localizations of mtFISH probe positions (see examples in Figs. 3 and 4).

**Figure 5:**
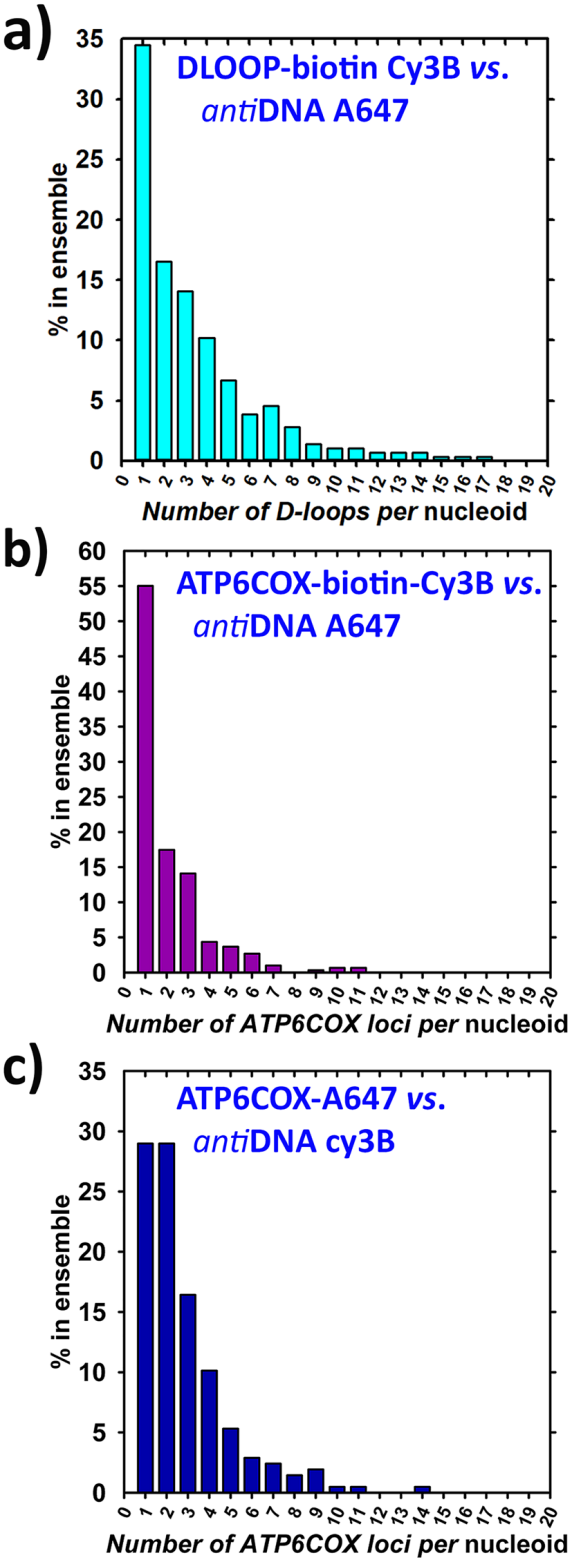
Histograms counting number of mtDNA (mtFISH *loci*) *per* individual nucleoids as % within a nucleoid ensemble. Protocols for combinations of mtFISH *vs*. nucleoid staining are ascribed to each histogram a–c in relation to Fig. 1a–c. mtDNA molecules *per* nucleoid were counted either as D-loops or *ATP6*-*COX3* gene regions. Numbers of reconstructed nucleoids with overlaps within ensembles were as follows: 284 (**a**), 298 (**b**), and 207 (**c**). These numbers were set as 100%. Histograms are based on 200 to 300 3D overlaps each and were derived from the 3D data matrix containing 20,000 to120,000 fluorophore blinkings (“points“) in a nucleoid channel. Biological replicates *N* were 3 for each histogram.

We have first calculated *n*^*i*^_mtDNA_ value count, representing the number of mtDNA-hybridized *loci* (D-loops or *ATP6-COX3 loci*) detected in each individual nucleoid. Next, we displayed for each n_mtDNA_ = 1, 2, 3, 4, … how many nucleoids (expressed in % of all imaged nucleoids) contained these n_mtDNA_ values and thus constructed the resulting histograms (Fig. 5a–c). The mtDNA-hybridized *loci* were taken in both cases, when visualized by a single or also by multiple fluorophores, blinking during the data acquisition period. In case of multiple blinking for a single spatial locus smaller than 80 nm (i.e. maximum mtFISH probe extension) the whole continuous encompassed spatial region was taken as a single n_mtDNA_, i.e. as a single location for the hybridized mtFIS probe. Thus in most of Fig. 3 panels, nucleoids with n_mtDNA_ = 3 or 4 are illustrated.

In case of a few or two single fluorophores apart by a certain distance, a false positive identification may arise. This happens, for example, when two fluorophores blink on a single hybridized probe. However, to consider predominant contribution of such a bias, one should assume all hybridized probes to be fully unwounded and extended as mentioned above. Hence, when detecting two n_mtDNA_ at the opposite side of e.g. ~ 300 nm nucleoid, it is unlikely that the 250 bp probe completely extending up to 60 nm (and together with Ab up to 80 nm) could span the whole 300 nm diameter nucleoid (neither its 900 nm hemisphere surface circumference). Note that histograms based on 200 to 300 3D overlaps were derived from the 3D data matrix containing 20,000 to120,000 fluorophore blinking (“points“) in a nucleoid channel (Fig. 5a–c).

Figures 3 and 5a represent a dual antibody staining (Figs. 1a, 2g). D-loops were imaged as the *loci* of DLOOP-biotin-Cy3B labeling, while nucleoids were also visualized using primary *anti*-DNA (IgM-based) antibodies and A647-conjugated secondary antibodies (“*anti*-DNA-A647”; blue in Fig. 3). Images for the acquired localizations of the 250 bp 7S-mtDNA-biotin hybridization probe (“DLOOP-biotin Cy3B”) were overlaid with nucleoids in 3D space. Note that despite visualization of the latter probe by antibody staining (including secondary Cy3B-560-conjugated antibodies), the visualized *loci* were on average much smaller than observed nucleoids. We did not discard any localization from the obtained 3D data. If any localization represented a drift or noise, the contribution of these effects does not significantly affect results derived from the obtained large statistical ensemble of the 3D point matrix. In contrast, if there were too many localized fluorophores for mtFISH and were expanded in a larger space, this case might reflect two or more adjacent *loci* unresolved by tessellation.

Our approach provided a sufficient statistics for construction of *n*^*i*^_mtDNA_ histograms (Fig. 5a). It involved preceding tessellation, i.e. segmentation of 3D data of both channels mixed, followed by the separate segmentation and Delaunay tetrahedron modelling of nucleoids^38^ and separate visualization of D-loops or other *loci* within the segmented overlaps. The histogram x-axis includes a stepwise-increasing *n*^*i*^_mtDNA_ values, meaning how many of mtDNA-hybridized *loci* were detected in each individual nucleoid. The y-axis shows in %, how many of such nucleoids existed within the nucleoid ensemble of all acquired double-stained images. In Fig. 5a, the number of D-loops encountered in a single nucleoid extended into values up to 17 *loci* (D-loops) *per* constructed nucleoid. Only 35% of constructed nucleoids contained exclusively a single overlapping D-loop region; 17% contained 2; 14% contained 3; 10% contained 4; 7% contained 5; and up to 5% contained 6 or 7 , while 3% contained 8 D-loop regions. More than 9 to 17 D-loop regions were found each only in less than 2% nucleoids (Fig. 4a). If we consider that any number over 6 is excessive and may originate from a bias, noise, or drift or perturbed localizations, still the weighted average calculated only with *n*^*i*^_mtDNA_ < 7 was 2.07. This is much higher than the previously reported value of ~ 1.4^27,28^.

**Figure 6:**
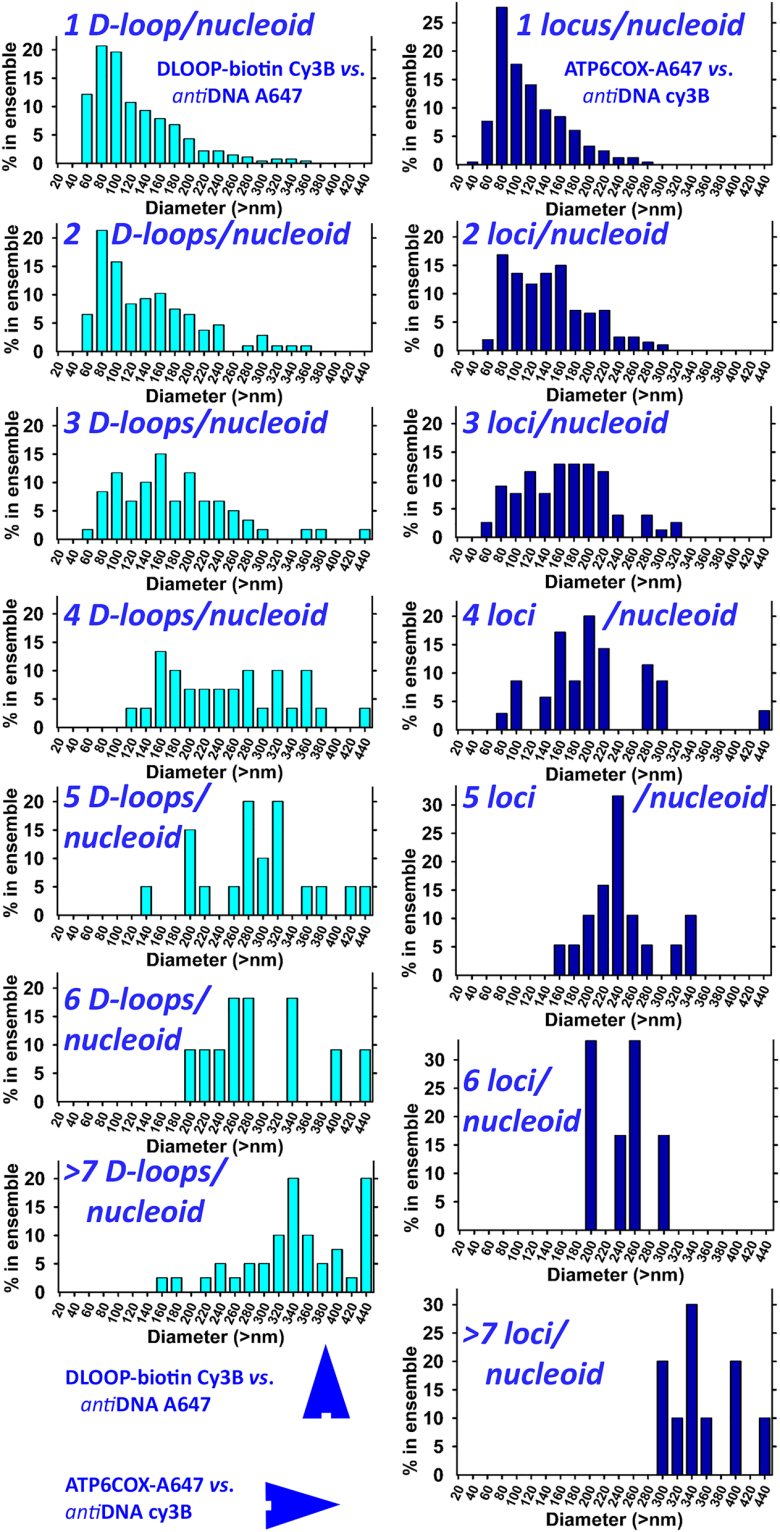
Histograms correlating number of mtDNA *per* nucleoid with the nucleoid diameter in HeLa cells—available data for protocol of “DLOOP-biotin Cy3B *vs*. antiDNA-A647” (*right panels*) and “ATP6COX-biotin Cy3B *vs*. antiDNA-A647” (*left panels*) were used for the displayed histograms, each one for different *n*^*i*^_mtDNA_, *i.e*. number of mtDNA *per* nucleoid, the last one for all *n*^*i*^_mtDNA_ equal or higher than 7.

We have also correlated the obtained nucleoid diameters with *n*^*i*^_mtDNA_ (Fig. 6, left panels). To this end, models of nucleoids with overlapped mtFISH *loci* were used, which might be slightly larger than those plain mtDNA nucleoid cores. The resulting constructed histograms of nucleoid diameters for *n*^*i*^_mtDNA_ = 1 to 6 and > 7 yielded a correlation showing that more D-loop regions or hybridized *loci* exist in larger nucleoids. This also excludes a high contribution of a bias from multiple blinking of a single probe. For increasing *n*^*i*^_mtDNA_ from 1 to 7, the resulting histograms shifted towards larger diameters, having the most frequent distribution value (MFD) around 80 nm with *n*^*i*^_mtDNA_ 1 or 2, increasing MFD to 160 nm with *n*^*i*^_mtDNA_ 3 or 4, and up to 300 nm with *n*^*i*^_mtDNA_ > 5. Because of a wide shape of these histograms, we could speculate that there exist small nucleoids with more than one D-loop. Nucleoids with twofold *n*^*i*^_mtDNA_ may represent those at instant division upon mtDNA replication for an original *n*^*i*^_mtDNA_ nucleoid. This complies with their higher mtDNA content present in higher volume. Nevertheless, as we demonstrate below, mtDNA expands and condenses depending on the recruitment of TFAM and other mtDNA expression machinery proteins, hence the immunostained volume of mtDNA core does not accurately reflect the DNA amount.

### Hybridization of *ATP6*-*COX3* region of mtDNA within HeLa cell nucleoids

Next we intended to relate the mtDNA count on another mtDNA region than the D-loop. To this end, we used the 1,100 bp hybridization probe spanning the overlapping *ATP6*-*COX3* region of mtDNA with inserted biotin (Figs. 4, 5b). This mtFISH probe was visualized by *anti*-biotin primary Ab followed by Cy3B-560-conjugates secondary antibodies (“ATP6COX-biotin-Cy3B”) (Fig. 1b). The mtDNA cores of nucleoids were simultaneously imaged using the “*anti*-DNA-A647” protocol. The resulting histograms of *n*^*i*^_mtDNA_ (Fig. 5b) were similar to those obtained for the DLOOP-biotin-Cy3B /anti-DNA-A647 combination of Fig. 5a. The weighted average of *n*^*i*^_mtDNA_ < 7 was 1.8, *i.e*. when neglecting multiple *loci* 7 and more. Despite one would assume a higher contribution of a bias due to multiple blinking of a single probe at two distant *loci*, the resulting histogram is extended to lower *n*^*i*^_mtDNA_, whereas the biased data should be extended to a false positive higher *n*^*i*^_mtDNA_.

In the third design (Figs. 1c, 2a–c,e,f, 5c, 6, right panels), we employed a similar 1,100 bp hybridization probe spanning the overlapping *ATP6*-*COX3* region of mtDNA, but with directly inserted A647-UTP (“ATP6COX-A647 probe “). Nucleoid mtDNA core was imaged using the “*anti*-DNA-Cy3B-560” protocol. Only 29% of constructed nucleoids contained exclusively a single overlapping *ATP6*-*COX3 loci*; 29% contained 2 *loci*; 17% contained 3 *loci*; 10% contained 4 *loci*; 5% contained 5 *loci*; and up to 3% contained 6,7,8 or 9 *loci*, while < 1% contained more than 10 to 14 *loci* (Fig. 5c). The weighted average of *n*^*i*^_mtDNA_ < 7 was 2.2, *i.e*. close to counting of D-loops in Fig. 5a. We observed the same shift in nucleoid diameter histograms with increasing *n*^*i*^_mtDNA_ (Fig. 6, right panels).

### Nucleoid size distribution in 143B osteosarcoma cells with mutant TFAM

**Figure 7:**
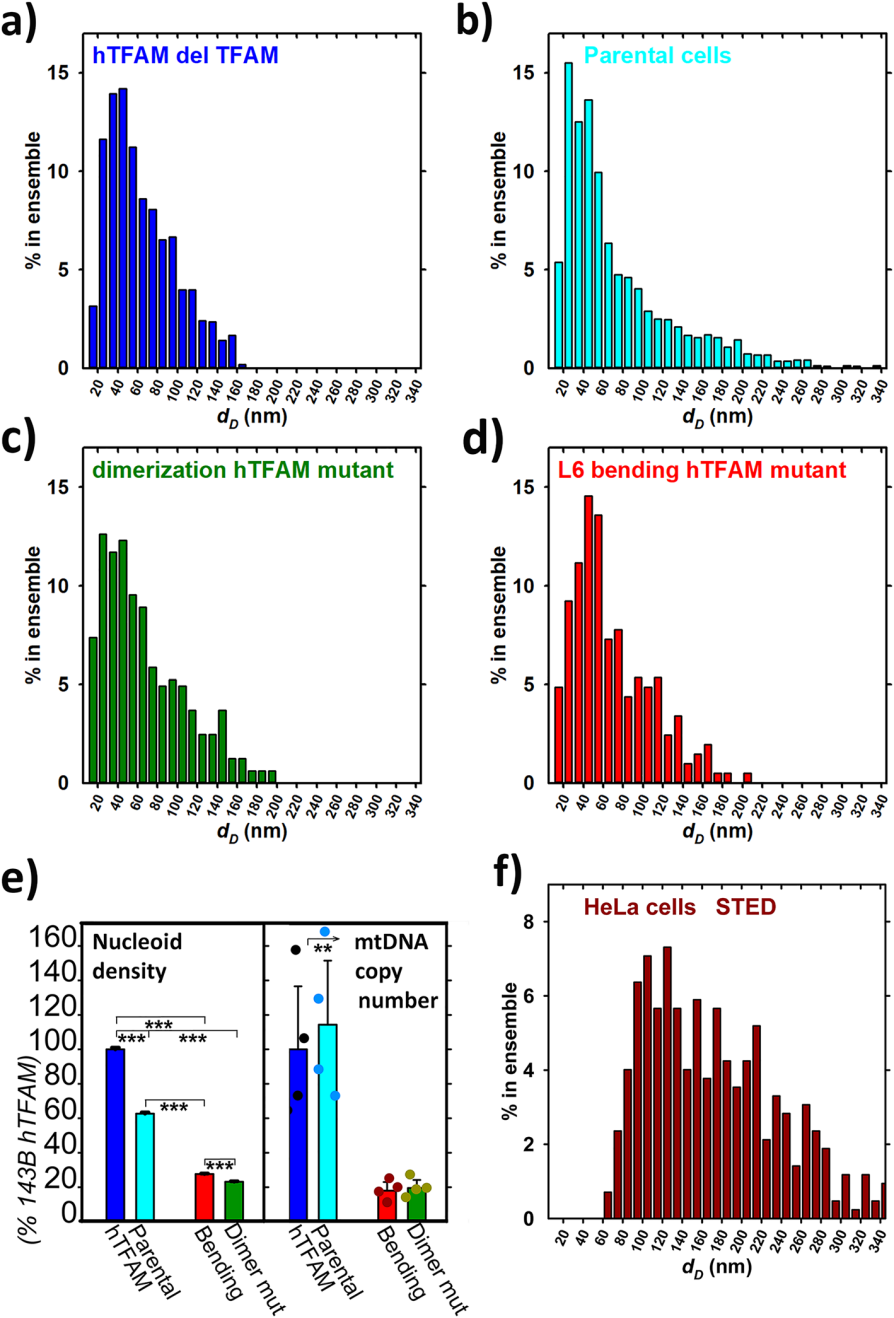
Nucleoid diameter distribution and density *vs*. copy number with mutant TFAM. TFAM mutant types for osteosarcoma 143B cells are ascribed to each histogram of nucleoid diameters for spheroid models (**a**–**d**). Graph in panel (**e**) shows nucleoid density for 134B cells and their respective TFAM mutant knockins (three samples for each), compared with the mtDNA copy number. ANOVA: ** *p* < 0.01; *** *p* < 0.001. Note, nucleoid density is used instead of nucleoid number *per* cell owing to the unknown cell volume. Panel f) shows a representative histogram of nucleoid diameters of HeLa cells immunostained with *anti*-DNA-A647 imaged by 2D STED microscopy (cf. Figure 2d).

To recognize different distributions of nucleoid diameters imaged by 3D dSTORM, we also varied human TFAM (hTFAM) to mtDNA stoichiometry, thereby altering mtDNA copy number and nucleoid size. For these studies, we employed human 143B osteosarcoma cells with mutant TFAM. Visualization of mtDNA nucleoids by *anti*-mtDNA 3D dSTORM in hTFAM-overexpressing *vs*. parental osteosarcoma 143B cells showed again a wide distribution of nucleoid diameters (now considering them as spheroids, see Materials and Methods), extending between 20–30 nm up to 160 nm *vs*. 280 nm, respectively (Fig. 7a,b). Despite the smallest objects found by tessellation/segmentation could be regarded as incompletely contoured, one may interpret the difference in having a higher fraction of 80–120 nm nucleoids and no appearing diameters over 160 nm after hTFAM overexpression by the TFAM ability to bend and crosslink more intensively mtDNA. This would result in packing it in a higher extent. The most frequent distribution, MFD, *i.e*. the peak in the distribution histograms of nucleoid diameters, was found to be nearly constant at about 50 nm.

Next, we evaluated knockin 143B cells expressing mutated hTFAM. Either, so-called dimerization hTFAM mutant was overexpressed, being unable to dimerize, hence unable to crosslink mtDNA chains; or an L6-bending mutant of hTFAM was overexpressed, which cannot bend mtDNA. Both mutations affected mtDNA replication and transcription, as reported previously^46,47^. When visualized by *anti*-DNA 3D dSTORM, 143B knockin cells overexpressing either one of hTFAM mutants exhibited similar nucleoid diameter distribution as the parental cells, reduced to diameters only up to 200 nm (Fig. 7c,d). This shows that unlike the overexpressed hTFAM, the dimerization mutants lose the ability to add nucleoids within the 80–120 nm diameter range. But paradoxically, the same is applied for the L6-bending mutant.

To evaluate effects of mutations relative to the amount of mtDNA within the cell, expressed as mtDNA copy number, we first derived the 3D spatial density of nucleoids (ρ_nucl_; Fig. 7e). We used this quantity instead of the total number of nucleoids within the cell, because of complications with determination of the cell surface that would require the third optical channel. It was no surprise to see that hTFAM overexpression led to ~ 1.67-fold higher ρ_nucl_. In contrast, both dimerization and L6-bending mutations led to drastic decrease of ρ_nucl_. Indeed, mtDNA copy number decreased by the same extent, *i.e*. down to ~ 20% (Fig. 7e). However, cells overexpressing hTFAM and having ~ 1.67-fold higher ρ_nucl_ exhibited mtDNA copy number equal to that in parental cells. Probably, hTFAM overexpression established such a steady state of mtDNA replication/mtDNA degradation, which only enlarged number of medium-sized nucleoids within the 80–120 nm but preserved the mtDNA total content in the cell. We can speculate that expansion of mtDNA by unpacking happened. By the other words, hTFAM overexpression led to a higher TFAM to mtDNA stoichiometry and to more nucleoids.

### Wide nucleoid size distribution is apparent also by STED microscopy

As examples in Figs. 2d and 7f demonstrate, non-uniform nucleoids are visualized also when using 2D-STED for *anti*-DNA-A647 nucleoid imaging in HeLa cells, despite this purely physical method is employed. The nucleoid projections in a confocal plane were analyzed and resulting diameters were estimated to be between 60 to > 300 nm, which matches not only 3D dSTORM data of two channels (cf. histograms of Fig. 6), but also previously published data with cultured cells^38,41^ as well as primary β-cells of pancreatic islets^40^.

### Independent pattern analyses based on 3D Ripley's K-function

**Figure 8:**
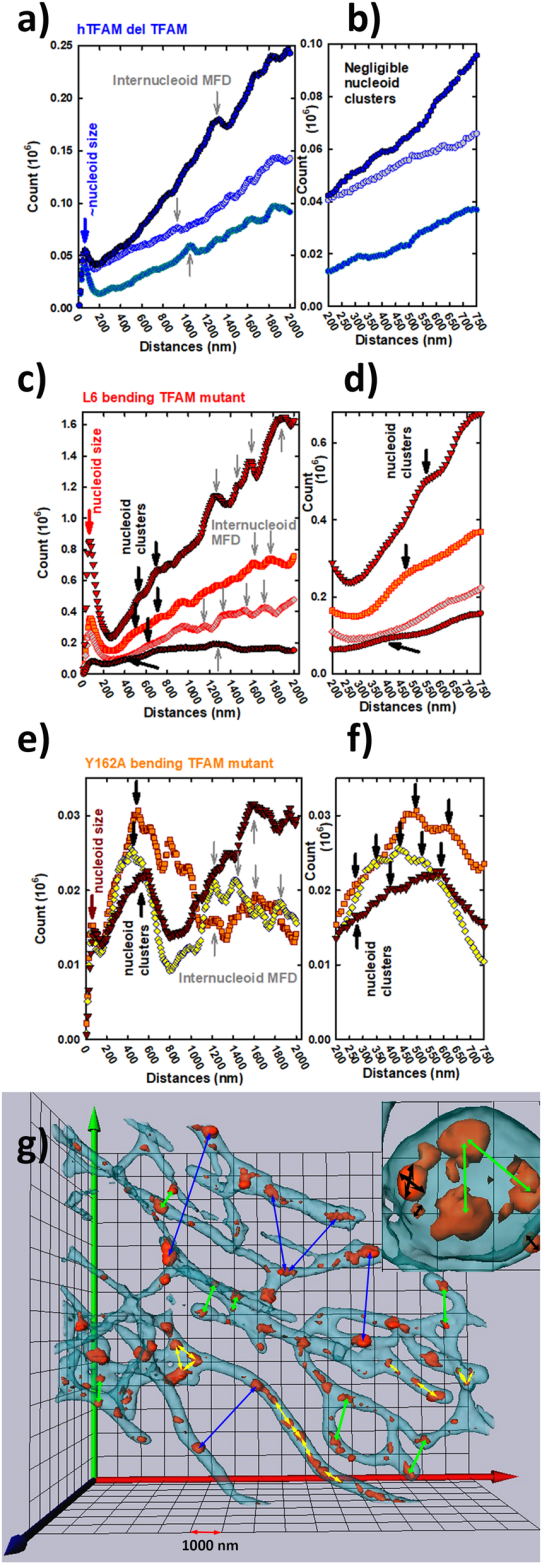
Histograms of all inter-distances between nucleoid-staining fluorophores in 134B cells. (**a**,**c**,**e**) full range of distances; (**b**,**d**,**f**) detailed medium distance range 200 to 750 nm. The inter-distance histograms were derived on the basis of Ripley’s K-function as described in Material and Methods. Software calculating Ripley’s K-function was a part of Vutara data acquisition package. (**g**) 4Pi microscopic 3D image of nucleoids within the mt tubular network (such as from Ref. 37) is shown for illustration of interdistances within a nucleoid (inset, black arrows), within a cluster of nucleoids (inset, green arrows), existing in bulky portions of network; and inter-nucleoid distances inside a single mt tubulus (yellow arrows) or its bulky part (triangles from yellow arrows; green arrows in inset) or distances between nucleoids of different tubules (those shorter than 2000 nm: green arrows, longer distances: blue arrows).

To illustrate even more the nucleoid clustering issue, an in-depths analysis of the original 3D dSTORM images was performed using the histograms of (inter)distances derived from the 3D Ripley's K-function (Fig. 8)^39,46^. Note that distances between each 3D-localized fluorophore blinking events are taken to the account here, to provide an unbiased analysis in contrast to predetermined, e.g. spherical, modeling of nucleoids. This analysis yielded three categories (ranges) of inter-distances, termed further simply as distances. An existence of a local peak (MFD) at 50 nm can be interpreted either as being the minimum limit of 3D distance resolution. However, it may reflect also the most frequent diameter of nucleoids. Since the identical most frequent diameter was found by the spherical modeling of nucleoids, we prefer interpretation that MFD from 3D Ripley's analysis originated also from the frequently repeating distances between fluorophores coating *anti*-DNA antibodies on the presumable nucleoid surfaces. Next, the distance distribution above this local maximum up to the subsequent local minimum can be taken as independently evaluated statistical distribution of nucleoid sizes (despite the realistic irregular surface shape differing from the spheroid).

The second category of distances is given by local peaks in interdistance-histograms within the range from 200 nm up to a realistic diameter of the mitochondrial network tubules (see enlarged parts of histograms in Fig. 8b,d,f). These distances and the local histogram maxima most probably reflect the existing nucleoid clusters, *i.e*. the individual distance spans from the fluorophore on a coating antibody of one of nucleoids within the cluster to another such fluorophore in a different nucleoid. Either lower distances than the average of 1000 nm exists between two nucleoids within a single mt tubule (Fig. 8g) or nucleoids concentrate in a cluster in bulkier spheroid portions of mt network (Fig. 8g, triangles by arrows and inset). The third category of local maxima finally corresponds to the inter-nucleoid distances (in histograms, inter-nucleoid MFDs; Fig. 8g), either between neighbor nucleoids of the same mt tubule or of different mt tubules.

A similar analysis of the obtained 3D dSTORM images of our three mutant types of cells with increased TFAM content *vs*. those having a specifically disrupted function of TFAM, we found the following features (Fig. 8). Cells with overexpressed wt hTFAM exhibited negligible clusters of nucleoids. Knockin cells overexpressing L6 bending hTFAM mutant contained clusters of one to two MFDs, but cells overexpressing Y162A bending hTFAM mutant contained clusters of more different sizes reflected by up to five MFDs.

## Discussion

We attempted to count the D-loops or *ATP6*-*COX3 loci* of mtDNA within nucleoids from stochastically localized fluorophores of mtFISH probes, in order to count how many mtDNA molecules exist in each individual nucleoid. Both 3D-dSTORM imaging and determination of 3D-spatial overlaps were stochastic, so to provide a certain statistics, despite the fact that images/overlaps might contain a certain fraction of a noise or imperfectly contoured objects and which might be biased by an unknown portion of a multiple blinking at the opposite distant *loci* of a single hybridization probe. When considering that such imperfections could lead to any number of overlaps equal to and higher than 7, we obtained a weighted average for *loci* number *per* individual nucleoid, *i.e*. number of mtDNA molecules *per* nucleoid around 2 in HeLa cells. This was the case when we used our best imaging modalities. Notably, it is higher than the previously reported value of 1.4, based on STED imaging [27;28].

Nucleoids were immunostained with *anti*-DNA Ab and their wide size distribution was analogical to distribution reported previously for mtDNA core of nucleoids^38^. In our cell samples we obtained non-uniform nucleoid size distribution even when using 2D STED for control. An automated procedure for finding 3D overlaps of two colors (channels) was employed, based on Delaunay tessellation, segmenting objects^38^. This procedure was first applied to mixed data of both channels followed by the individual tessellation/segmentation of each channel separately. Since nucleoids were imaged in one of the optical channels, data of this were approximated by ellipsoids, as published elsewhere^38,40^. A simple counting of mtFISH overlapping regions was taken to reflect the number of mtDNA molecules in each nucleoid. We succeeded with mtFISH probes against D-loops or desired mtDNA sequences of *ATP6*-*COX3 loci*. From the obtained statistical ensembles, we demonstrate that 45% to 70% of visualized nucleoids, contained two or more D-loops or *ATP6*-*COX3 loci*, when entire nucleoids were stained with *anti*-DNA (Fig. 5a–c). With biotin mtFISH probe, still a sufficient fraction of 45% nucleoids contained two or more mtDNA molecules (Fig. 5b).

Those nucleoids with *loci* number (mtDNA molecules) *per* individual nucleoid of 2 or 2n (n > 2) may represent nucleoids, where mtDNA replication just proceeds. This was previously indicated by “snapshot” of 3D super-resolution stochastic microscopy enabled in fixed cells^38^. These nucleoids would be therefore in a state prior to their division. Nevertheless, both used techniques (our 3D dSTORM *vs*. 2D STED;^27,28^) suggest that it is quite rare when more than 4 mtDNA exist in nucleoids. In conclusion, our data suggest that frequent multiple mitochondrial DNA copies may exist in a single nucleoid in HeLa cells.

We also demonstrated that the TFAM-induced nucleoid size expansion can happen at constant mtDNA, even when mtDNA is visualized. Thus, TFAM overexpression may influence nucleoid restructuring. TFAM is also a major factor determining mtDNA copy number within the cell. The used osteosarcoma 143B cells with overexpressed human TFAM increased 1.67-fold their nucleoid spatial density (the parameter used instead of the total number of nucleoids within the cell) at virtually unchanged mtDNA copy number. At the same time, a fraction of 80–120 nm nucleoids increased, meaning number of nucleoids increased at constant amount of mtDNA, indicating dilution or expansion of existing mtDNA and its redistribution into more nucleoids upon the higher TFAM to mtDNA stoichiometry.

Also, it shows that the size of nucleoids is not dependent exclusively on a number of mtDNA molecules within the nucleoid, but is rather determined by the content of TFAM (and possibly by other recruited proteins) or more exactly by TFAM to mtDNA stoichiometry. The recruited TFAM to a sufficiently packed mtDNA could even increase the size of the nucleoid, simply because of the additional TFAM mass. In contrast, upon drastic reduction of mtDNA copy number down to 20% after overexpression of either bending or dimerization TFAM mutant, the nucleoid spatial density decreased by the same extent. This reflects that the inherent optimum TFAM/mtDNA stoichiometry exists.

### Limitations of the study

We are aware of some uncertainties inherent in our developed method. Their character is either biological, it may originate from an imperfect design of the method or uncertainties might be photophysical. Concerning nucleoid biology, we admit that imaging of immunostained nucleoids in parallel with the hybridization technique could distort their original native size or shape due to the required RNAse and DNase I pretreatment, but cannot lead to clustering, which proceeds in bulky mt network spheroids^37^. Thus attempting a high precision in localization, the absolute dimensions might be imprecise. Immunostaining itself, including the secondary antibody staining, covers real shapes of biological objects by up to ~ 20 nm thick layer because of Ab dimensions^33^. We may consider that such imprecision extends sizes of overlapping entities equally and affects only the absolute sizes of objects (nucleoids), hence it does not affect the existence of overlaps as such and character of size distribution. We thus demonstrate again in this work, that independently of the staining and notably also independently of the imaging method, nucleoids always have a range of sizes. A weak relationship between a higher number of D-loops and larger size of those nucleoids was found (Fig. 6). As a result, we have encountered smaller nucleoids having possibly more than one D-loop, medium-sized nucleoids with more D-loops, as well as larger nucleoids with a single D-loop. In the future, comparison of cells with elevated vs. compromized mtDNA replication would elucidate whether 2n mtDNA molecule numbers per nucleoid result from mtDNA replication and yet unseen nucleoid division.

An imperfect design of the method lies in the impossibility to have a reasonable signal for dSTORM from a single-fluorophore containing hybridization probe. This might introduce some bias for smaller (or incompletely visualized) nucleoids due to possible multiple blinking at the opposite distant *loci* of a single hybridization probe. As noted above, this bias should not be dominating in the data sets, especially for 250 bp probe and medium-size or large size nucleoids.

Certain errors may also stem from our developed procedure of D-loop or *loci* counting, specifically from the way that we did not discard any single localization of fluorophore. We may admit that if these localizations are false positive, *i.e*. being represented by a noise or the above mentioned multiple blinking of a single probe, than the *loci* are counted as *n*^*i*^_mtDNA_ + 1 in this particular nucleoid. Nevertheless, within the obtained larger statistical ensemble, these errors cannot change the overall derived pattern. In contrast, yet another drawback coming from the insufficient resolution may now underestimate *n*^*i*^_mtDNA_. Typically, when several localizations for the D-loop (7S) or *ATP6*-*COX* fluorophore were in a close contact, we took this locus as a single one. For a lower number of localizations this reflects the reality of having more fluorophores within the probe, possible drift, etc. However, when for example 50 localizations spread in a substantial space distance is taken after Delaunay tetrahedron modeling as a single locus, it may be false negative, since in reality there could be two D-loops (sequence parts) instead of the counted one.

## Conclusions

We conclude that frequently more than one mtDNA molecule exist within a single mitochondrial nucleoid in HeLa cells. In some cases 2n number of mtDNA molecules in a single nucleoid may reflect situation of mtDNA replication just instantly prior to the putative nucleoid division. We also demonstrate that size of nucleoids is not dependent exclusively on a number of mtDNA molecules within the nucleoid, but is rather determined by the content of TFAM.

## Methods

### Cells

Human cervix epitheloid carcinoma HeLa cells (ECACC 93021013; RRID:CVCL_0030) were cultured at 37 °C in Dulbecco′s Modified Eagle′s Medium (DMEM) with 3 mM glutamine, 10% (v/v) fetal calf serum (FCS), 10 mM HEPES, 100 IU/ml penicillin, 100 μg/ml streptomycin, and 5 mM glucose in humidified air with 5% CO_2_. Human osteosarcoma 143B cells were purchased from American Type Culture Collection (ATCC) (CRL-8303™; RRID:CVCL_2270) and 143B cells containing various TFAM mutant forms were a kind gift from Dr. Mikhail F. Alexeyev (University of South Alabama, 307 University Blvd, Mobile, Alabama, 36688, USA). The 143B cell variants were as follows: *i*) parental 143B cells; *ii*) 143B^hTFAM^ cells, in which endogenous TFAM has been inactivated and wt hTFAM cDNA was re-expressed; *iii*) 143B^Y162A_hTFAM^ cells, in which endogenous TFAM has been inactivated and Y162A hTFAM bending mutant was re-expressed; *iv*) 143B^L6_hTFAM^ cells, in which endogenous TFAM has been inactivated and L6 hTFAM bending mutant was re-expressed. Cells were cultured in DMEM containing 10% FCS and 50 µg/ml gentamycin, which was optionally supplemented with 50 µg/ml uridine and 1 mM pyruvate.

### Estimation of mtDNA copy number (C_*N*_*)*

mtDNA was isolated by phenol/chloroform extraction. Primers annealing on the *UCP*2 nuclear gene (intron 2 and exon 3) and the *ND*5 mitochondrial gene (11092–11191 bp) (sequences from The National Center for Biotechnology Information, USA) were used for SYBR Green qPCR amplification. Alternatively, 7S mtDNA sequence (15412–16309 bp) was used to detect mtDNA with deletions. The ratio between the *ND*5 amplicon (7S mtDNA) and half of the nuclear amplicon amounts was calculated as the mtDNA copy number *per* cell (*C*_N_).

### Control imaging using STED

Employing DNA immunostaining (see below), resulting samples were imaged by STED microscopy on an inverted microscope DMi8, equipped with laser scanning confocal head Leica TCS SP8 (Leica, Mannheim, Germany) and differential interference contrast for all objectives, at the Institute of Molecular Genetics of the Academy of Sciences (Prague) core facility. The confocal head was equipped with two fluorescence PMT detectors and two highly sensitive HyD detectors with time resolved gating function. The STED 3X module was equipped with a 660 nm continual depletion laser, with a specification of 35 nm x,y-lateral, 130 nm z-axial resolution. Actual resolution of acquired 2D x,y-images was worse (cf. Figure 2 legend). Analyses of STED-visualized nucleoid diameters were performed using the CellProfiler software (https://cellprofiler.org/; RRID: SCR_007358).

### mtDNA fluorescence in situ hybridization (mtFISH) protocol

5-ethynyl-dUTP (EdUTP) was used instead of dTTP in PCR and the resulting PCR yielded products with proper fluorophores, while biotin was introduced by „Click-it “ azide-ethynyl chemical reaction using kits from Jena Biosciences (Jena, Germany). Fluorophore-containing mtFISH probes were prepared by PCR using Alexa Fluor 647-dUTP (A647-dUTP) from the HighFidelity AF647 PCR Labeling Kit (Jena Bioscience). Cells were fixed using 2% paraformaldehyde in a shaking incubator at 37 °C for 10–15 min. Afterwards, cells were washed in phosphate buffered saline (PBS) three times on a shaker at room temperature for 10 min. To remove single strand RNA (ssRNA), RNase A (Merck, Darmstad, Germany) treatment was performed in PBS at 37 °C for 2 h under constant agitation. To degrade RNA from RNA**/**DNA-hybrids, RNase H (Merck) digestion was applied at 37 °C for 30 min under constant agitation. To digest dsDNA, DNase I (Merck) treatment was performed 37 °C for 30 min under constant agitation. DNase I and RNase H treatments were performed in the reaction buffer prepared according to the manufacturer’s protocol.

Coverslips (precision cover glasses thickness No. #1.5H (tol. ± 5 µm), 22 × 22 mms; Marienfeld, Germany) were checked for a uniform thickness and washed using 0.05% Triton-X in PBS at room temperature for 10 min under constant agitation. After the resulting permeabilization, coverslips were *pre*-hybridized in 50% formamide with 10 µl of salmon sperm DNA in 2ˣSSC buffer at room temperature for 30 min under constant agitation. Subsequently, samples were incubated with 70% formamide containing 10 µl of salmon sperm DNA in 2ˣSSC buffer at 72 °C for 10 min. 100 ng of the PCR probe in 50% formamide with 400 µl of 25% dextran sulfate and 2 µl of salmon sperm DNA were added onto the coverslips after 10 min of probe denaturation at 80 °C and hybridized at 37 °C for 12 h under constant agitation. Coverslips were then washed three times in PBS at room temperature. For double channel (color) dSTORM (see below), the 3D immunostaining followed. When attempted in the opposite order, in situ hybridization did not work.

### 3D immunocytochemistry for dSTORM

The 3D stochastic mode of super-resolution microscopy, direct stochastic optical reconstruction microscopy (dSTORM), was employed for a double-channel (color) acquisition^42^. Nevertheless, trials using a single-channel (color) acquisition were done to check the protocols. Cells were cultured on #1.5H coverslips (Marienfeld, Germany) coated with poly-l-lysine. The coverslips and were carefully checked for a uniform thickness. Cells were then fixed using 4% paraformaldehyde for 10 min and washed twice in PBS).

For double-channel acquisition the protocol described below followed in situ hybridization. Further steps were done in “TxPBS” containing 0.05% Triton X100, 0.05% Tween 20, and 0.1 M glycine. The fixed and/or hybridized cells were blocked with 5% donkey serum (Jackson ImmunoResearch, West Grove, PA, USA) for 1 h and then either Alexa Fluor 647-conjugated *anti*-TFAM primary antibody (Abcam, Cambridge, MA, USA; Cat# ab198106) was used; or unconjugated primary *anti*-DNA (IgM-based; Progen Biotechnik, Heidelberg, Germany, Cat# 61014; RRID:AB_2750935) or *anti*-biotin (Abcam, Cat# ab53494; RRID:AB_867860) were used. For Fig. 2h also rabbit *anti*-mtSSB (Sigma, St Louis, MO, USA) and rabbit *anti*-FIS1 (Proteintech, Rosemont, IL) antibodies were used. Coverslips were then washed three times in “washing PBS” and incubated with donkey *anti*-mouse IgG secondary antibody conjugated with Alexa Fluor 647 (Thermo Fisher Scientific, Cat# A-31571; RRID:AB_162542) and similarly, *anti*-rabbit IgG A647-conjugated (Thermo Fisher Scientific, Cat# A-31573; RRID:AB_2536183) or our own prepared IgG secondary antibody conjugated with Cy3B, using the Cy3B NHS ester (GE Healthcare, Pittsburgh, PA, USA; Cat# PA63101). Finally, samples were washed three times in PBS and mounted in the “dSTORM buffer”, i.e. 10 mM NaCl, 50 mM Tris–HCl (pH 8.0), containing 10% glucose, 50 mM β-mercaptoethanol, 169 units of glucose oxidase, and 1.4 units of catalase. Only stained cells were imaged.

Imaging was performed on a biplane-mode fluorescence photoactivated localization microscopy (FPALM) instrument^38–40,48^, a prototype of Vutara (formerly Vutara, Salt Lake City, UT, USA; now Bruker Nanosurface, Middleton, WI, USA), equipped with lasers (Coherent, Santa Clara, CA, USA) emitting at 405 nm (Obis 405), 488 nm (Sapphire 488–200), 561 nm (Sapphire 561–200) and 641 nm (Cube 640–100). Light from lasers was collimated and sent through an acousto-optic tunable filter (AOTF) (Gooch & Housego, Ilminster, UK; R64040) to a multimode fiber. Emerging light from the fiber was reflected by a multiband dichroic beamsplitter (Semrock, Rochester, NY, USA; Di01-R405/488/561/635) onto the back aperture of a 60 × /1.2NA UPlan-SApo 60 × /1.2w water immersion objective (Olympus, Center Valley, PA, USA) mounted on a P-725 PIFOC piezo objective scanner (Physik Instrumente, Karlsruhe, Germany). The collected fluorescence from the sample was filtered using a bandpass FF01-446/523/600/677-25 emission filter (Semrock) and split in front of an Evolve 512 EM-CCD camera (Photometrics, Tuscon, AZ, USA) to create two images of different focal planes^36^. Recorded raw images were localized and then analyzed by the Vutara SRX software package using established algorithms^38–40,48^. Localized data were subjected to accuracy-based filtering and a drift correction^36^. Both channels were spatially aligned via Vutara SRX SW^50^ calibration routine using 100-nm Tetraspec beads (Invitrogen)^49^.

Two raw images (512 × 256 pixels, 16 bit) were taken at 33 frames *per* second. A647-stained samples were imaged at 641 nm, 3.5 kW/cm^2^, while Cy3-B-stained samples at 561-nm and 5 kW/cm^2^. The average photon count from a single detected and localized signal was in the range between 1400 and 2300 depending on the staining used. The typical number of events *per* frame was around 1. To ensure an even distribution of detected molecules over the entire axial range, the objective position was stepped at 500 nm intervals over a range of about 3 μm for the selected cell. The axial scanning procedure was therefore repeated several times with only a fraction of the molecules activated during each cycle. This ensures nearly constant particle yield over the complete axial range. Measurement took place over the course of several minutes and resulted from ten up to ~ 500 of localized fluorophores *per* nucleoid (~ 1500 for single channel acquisition only). The average photon count from a single signal detected and localized was 400. The average number of events *per* frame was 6.2. Imaging of Cy3B preceded imaging with A647.

### Single nucleoid determination from the acquired 3D data

3D image reconstruction was done as described elsewhere^38,40^. ParaView software, version 4.3.1, 64 bit^51^, (RRID: SCR_002516) was used including Python programming language modules for tetrahedron parameter functions, thus ensuring filtering or drawing of the bounding ellipsoid. Routinely, only nucleoids constructed from more than 10 localized A647 (Cy3B) fluorophores were considered in each image. Sets of localized particles were segmented into individual nucleoids by the Delaunay 3D-triangulation algorithm^38,40^. The resulting convex hulls were filled with the corresponding tetrahedrons (pyramids) with the base size *A*_max_ and expressed in nm. Tetrahedrons with all edges shorter than the threshold value *A*_max_ represented rough nucleoid models, where an individual nucleoid is visualized by polyhedrons composed of a set of connected tetrahedrons. For *spherical modelling*, the resulting polyhedrons were approximated by spheres of equal volume as either *i*) the resulting polyhedron for each nucleoid (volume *V*_D_ yielding the corresponding diameter *d*_D_); or *ii*) smoothed polyhedron with tetrahedrons added to obtain convex shapes (volume *V* yielding the corresponding diameter *d*). *Ellipsoid modelling* was performed as a means of further refinement based upon the principal component analysis. Rotational ellipsoids were illustrated with a volume equal to *V*_D_ (for detailed description see^38^), yielding the diameter of the longest and the shortest ellipsoid axis *d*_max_ and *d*_min_. We have also taken account of ellipsoid orientation, defined by unit space vectors oriented along the longest ellipsoid axis. Their axial projections are denoted as *a*_x_, *a*_y_, *a*_z_. Defining θ as the angle between the longest ellipsoid axis and z axis, we have filtered out mtDNA nucleoids oriented with the longest axis tilted more towards the x,y plane as those with θ < 4. Assuming θ = *arctg* [(*a*_x_^2^ + *a*_y_^2^)^1/2^ / *a*_z_], the ratio |*A*_*r*_**/a**_*z*_| has to be higher than 1 for θ < 45°.

### Analysis for 3D overlaps of dSTORM imaged mtDNA fluorescence hybridization probes with nucleoids

A two-step procedure based on the 3D Delaunay tessellation and subsequent modeling by principal component analysis was used to achieve segmentation and 3D rendering of overlapping of nucleoids and objects given by mtFISH staining of mtDNA (Supplemental Information Part I, Fig. S1–S7). At first, 3D Delaunay tessellation/segmentation and subsequent modeling by principal component analysis^38^ was applied for combined data of both channels, *i.e.* for example of “nucleoid” and “D-loop” channel (Fig. 1d). The second step involved 3D Delaunay tessellation within the objects segmented in the first step, but separately for each channel (color). In this way, single nucleoids were segmented containing attached or inserted regions of visualized mtDNA hybridization probes. For each visualized nucleoid within the ensemble of nucleoids from each cell, the number of mtDNA regions was counted and taken as the number of mtDNA molecules within the particular single nucleoid (*n*^*i*^_mtDNA_; *i* = 1 to N, where N is the number of visualized nucleoids within the 3D image). ParaView software^51^ was used including our own created modules of Python programming language scripts: M1: _csvNucleoidsKmeansSurf2chSep440.pvsm; M2: ShowNucleotidSelectedComponentsSurf.py; M3: WriteNucleotidComponentsSurf.py; and M4: AutomateNetwork FISH Double Single Color.py. Their detailed description as well as the whole 3D overlap finding procedure is described in the Supplementary Information (Part I, Fig. S1–S7).

In the first step (M1 and M2), data of both channels were mixed and our previously published method^38^ was applied, segmenting objects based on Delaunay triangulation in 3D space, followed by facet culling. The polyhedron parameter *A*_max_ of 80 nm was applied. Resulting polyhedron objects yielded from tessellation were smoothed and were remodeled either by spheres (yellow spheres in Figs. 3 and 4) or by ellipsoids with volumes equal to the volume of the polyhedrons.

In the second step, we identified and distinguished data of *channel* 1 and *channel* 2 within the mixed objects found in the first step, using module M3 (Table S1). Thus we identified and distinguished data for each channel separately. For that we refined Delaunay tessellation with *A*_max_ equal to the most frequent diameter of the channel desired for nucleoids. Then we identified the overlapping regions with the other (second) channel. We took into account even the rare situation when a single point was localized in the 2nd channel. We generated a binding ellipsoid by using the smallest possible *A*_max_ for the second (overlapping) channel. Finally, we counted how many of such overlapping regions (binding ellipsoids) of the second channel existed within a single object (nucleoid) of the first channel. During this procedure parameters are yielded as summarized in the Table S2.

### Histogram analysis of 3D inter-distances between antibodies

The 3D inter-distance histogram can be derived on the basis of 3D Ripley’s *K*-function as outlined below. A cumulative frequency function (cumulative distribution function) *H*(*r*) is defined as the sum for all distances between points shorter than *r*:

1 $$ H\left( r \right) = \sum \sum_{{{\text{i}} < {\text{j}}}}^{{\text{n}}} d_{{{\text{ij}}}} = \raise.5ex\hbox{$\scriptstyle 1$}\kern-.1em/ \kern-.15em\lower.25ex\hbox{$\scriptstyle 2$} \sum \sum_{{{\text{i}} \ne {\text{j}}}}^{{\text{n}}} d_{{{\text{ij}}}} , $$

where *d*_ij_ < *r*_max_ are distances between points *i* and *j* (note that when *d*_ij_ is identical to *d*_ji_ and *i* = *j* those distances are excluded). The limit *r*_max_ exists, corresponding to *H*(*r*) = *N*^*r*max^, in which all distances are counted. The number of all distances for the existing number of points *n* and *r*_max_ is simply the longest distance between points: *N*^*r*max^ = *n* (*n* − 1)/2.

The 3D Ripley’s *K*-function then expresses the expected number of events (points) within the distance *r* from a randomly selected point^47^. Let’s assume a volume of interest in which *n* points (events) exist with distances *d*_ij_ (point *i*, point *j*). 3D Ripley’s *K*-function can then be expressed for the value *K*(*r*), denoting how many points become encircled within a sphere of radius *r*^48,52^:

2 $$ K\left( r \right) = V\cdot\;n^{ - 2} \cdot\;\sum \sum_{{\,{\text{i}} \ne {\text{j}}}}^{{\text{n}}} \delta_{{{\text{ij}}}} , $$

where the indicator function δ_ij_ = 1 if distance *d*_ij_ (point *i*, point *j*) ≤ *r*, otherwise δ_ij_ = 0.

Comparing *K*(*r*) to a completely spatially random case, for which *K*_*CSR*_(*r*) = (4/3)*·*π* ·r*^3^ (*i.e*. a volume of a sphere of radius *r*), the *L*-function normalized for the average point (event) density is defined as:

3 $$ L\left( r \right) = (3K\left( r \right)/4\pi )^{1/3} $$

This can be used to compare the calculated *L*(*r*) *vs*. the chosen diameter *r*. As a result, the meaning of the *L*(*r*)—r function is that a cluster value is ascribed to every voxel of a volume *V*, while such a value is relative to the expected case of a completely spatially random case. When values *L*(*r*)—r > 0 occur, clustering is indicated; when *L*(*r*)—r < 0 the regular (ordered) pattern exists. Obviously, when *L*(*r*)—r = 0, the situation indicates a completely spatially random case.

The Ripley’s *K*-function is related to the cumulative frequency function *H*(*r*) as follows:

4 $$ K\left( r \right) = 2\cdot\;V\cdot\;n^{ - 2} \cdot\;H\left( r \right), $$

since the number of events and the number of distances differ only in that the Ripley’s function counts them twice. Consequently, the histogram of (inter)distances described by the function *H*(*r*) can be derived from the 3D Ripley’s *K*-function using the consideration of scanning within the 3D space. For each point *k*, the list of distances centered on *k* is gathered and sorted so that distances are shorter than *r* and counted to obtain their sum *L*_*k*_(*r*). Scanning over all points (summation over all points *k*) for *r* ≤ *r*_max_, the cumulative function *L*(*r*) is obtained. To avoid data explosion, a table can be constructed of *L*(*r*) values *vs*. a stepwise *r*, with a step of ε. The resulting table will thus contain values given by *L*(ε), *L*(2 ε),…*L*(nε  = *r*_max_) plotted *vs*. ε, 2ε, 3ε,…nε = *r*_max_. Subsequently, a *histogram of distance frequency distribution* with a bin of length ε can be constructed. As a result, we can plot differences {*L*(nε = *r*_max_) − *L*(nε-ε)}; {*L*((n-1)ε) − *L*((n-1) ε-ε)}; … {*L*(2ε) − *L*(ε)} *vs*. {nε}, {(n-1)ε}, …{2ε}. In 2D, this procedure amounts to scanning an image using a circular ring (annulus) with a thickness of ε. For 3D images this is represented by scanning the 3D image as a spherical shell with a thickness of ε. Because of the relation of eq. {4}, such a histogram should provide the same local maxima as the histogram of plain inter-distances *d*_ij_ within the sphere *r*_max_.

Calculations of the 3D Ripley’s *K*-function (*L*(*r*) function) from the localized points by dSTORM imaging were done using the software package Vutara^39,46^. Adopting the above-described procedure, histograms of plain inter-distances between *loci* occupied by the chosen antibody molecules were constructed. Usually most frequent distribution values, *i.e*. maxima of histogram (MFDs), were judged from the local maxima existing in the resulting histograms that ordered all the inter-distances between the localized single antibody molecules. Either a single MFD was listed to characterize each image; or a detailed comparison between the two histograms for 3D images of two different samples was used by dividing the first histogram by the second one. The resulting variation function was plotted for all combinations comparing two series of 3D images.

## Supplementary Information

Supplementary Information.

## Data Availability

The data sets supporting the current study are available on request from the corresponding author, P.J. The description of the entire computerized procedure and calculations, including modules M1 to M4 of Python programming language, are included in the Supplemental Information.

## Abbreviations

Ab: Antibody
A647: Alexa Fluor 647
C_N_: Copy number (of mtDNA)
dSTORM: Direct stochastic optical reconstruction microscopy
EdUTP: 5-Ethynyl-dUTP
FISH: Fluorescence in situ hybridization
MFD: Most frequent distribution (value of)
mt: Mitochondrial
*n*_mtDNA_: Number of mtDNA molecules within a single nucleoid
STED: Stimulated emission depletion (microscopy)
TFAM: Transcription factor A, mitochondrial
