# Supplementary material for: Possible frequent multiple mitochondrial DNA copies in a single nucleoid in HeLa cells
Source: Sci Rep. 2023 Apr 8;13:5788. doi: 10.1038/s41598-023-33012-6 (PMC10082775; doi:10.1038/s41598-023-33012-6)
Supplement: Supplementary file 1 — Supplementary Information. [file 41598_2023_33012_MOESM1_ESM.pdf]

SUPPLEMENTAL INFORMATION

PART I OVERLAPPS OF TWO CHANNELS IN 3D SPACE - BY DELAUNAY TESSELATION

Two channel FPALM/dSTORM analysis of 3D data

Data analysis of 3D images in a single channel extended to two channels

A single channel 3D data obtained from stochastic 3D nanoscopy were analyzed based on Delaunay tetrahedron modeling of nucleoids, as described elsewhere [38]. Analysis employed ParaView software 4.3.1, 64 bit [50] (www.paraview.org; RRID: SCR_002516), including own created modules of Python programming language [38]. Figures S1a–c illustrate outcome of such analysis for a single nucleoid (Fig.S1a), joined analysis of two neighbor nucleoids (Fig.S1b), and analysis of two neighbor nucleoids, but each separately (Fig.S1c). To find overlaps in 3D space, the same principle as in Fig.S1b was used, when the data of the two channels were combined. After tessellation of proximal objects between the two respective channels, each channel in the found overlap was treated separately.

Single channel analysis for nucleoids included:

At first, we found all nucleoids (clusters of nucleoids), without taking into account the channel, (*ProbeNo*) using Delaunay tessellation with A_{max} =80 nm (A_{max} is the longest distance in tetrahedron). We calculated volume, ellipsoidal axes and other parameters [38]. We draw a sphere (*yellow*) of the same volume as volume of a sum of all tetrahedrons from Delaunay tessellation for a cluster (*skeleton*) of nucleoid (localized) points. Alternative ellipsoid modeling formed an ellipsoid having the same volume (*gray*). Figure S1a illustrates a sum of tetrahedrons (*blue*) encompassing the localized points (red spheres) forming a nucleoid 3D image, which has volume *RegVol*. Figure S1b shows a skinned skeleton of cluster of localized points forming a nucleoid 3D image, which has volume, termed simply as parameter *Volume* and so-called skin *Surface* was calculated.

Python module ***WriteNucleotidComponentsSurf.py*** was used for the above calculations and drawings yielding outputs described in the Table S1. Typically two csv files were created, the first one with basic data on tessellated objects and drawings and calculations for each cluster.

Double channel analysis for nucleoids/localized point clusters included:

From each cluster of proximal nucleoids (sub-clusters for localized DNA *loci*), we selected only points with the first channel (*Probe*=0). Using Delunay tessellation with this, we found the “sub-nucleoid” 0 after Delaunay tessellation. When we encountered localized points with the first channel (*Probe*=0) in a larger distance than A_{max}=80 within the data, there could be a varying number of such “sub-nucleoids” (sub-clusters for localized DNA *loci*), from none to several separate sub-nucleoids (sub-clusters). The same we did for points with the second channel (*Probe*=1). For each sub-nucleoid (sub-cluster) we calculate volume (*VolumeProbe1*, *VolumeProbe0*) and other characteristics of data (see ***WriteNucleotidComponents2ch.py output***). We also included other more distant but still proximal clusters (*translucent green*). Typically we did this for distance up to 500 nm. The procedure is described in Figure S2 and Table S2.The modules used were as follows.

File	Description
_csvNucleoidsKmeansSurf2chSep440.pvsm	The Paraview network file
ShowNucleotidSelectedComponents2ch.py	Macro to Show selected nucleoid
WriteNucleotidComponents2ch.py	Macro to Write csv and jpg for each nucleoid in experimental data file
AutomateNetwork FISH Nikol 151202- 2ch.py	Batch script to analyze experimental data files.

Table S1. **Output of the WriteNucleotidComponentsSurf.py module.**

Component_Tif	Image of identified nucleoid/cluster of localized points without identification of the probe (*i.e.* for *probe*=0 and *probe*=1 together)
Component_Pos_Tif	Image including localization in the 3D space
RegionId	Identification number of nucleoid/cluster of localized points
X	X-Coordinate for centre of gravity for nucleoid/cluster of loc. points
Y	Y-Coordinate for centre of gravity for nucleoid/cluster of loc. points
Z	Z-Coordinate for centre of gravity for nucleoid/cluster of loc. points
NumPts	Number of localized points
NumTets	Number of tetrahedrons found by Delaunay tesselation
Volume	Larger (wrapped) volume of nucleoid/cluster of loc. points
Radius	Radius of sphere having the volume equal to *Volume*
Surface	Corresponding surface of larger (wrapped)
RegVol	Volume equal to sum of cluster of Delaunay tetrahedrons (no wrapping, *i.e.* smaller volume)
Delunay Avg radius	Radius of a sphere with volume equal to *RegVol*
Volume per hexagon	*Volume*/*NumTets* – average volume of hexagon (tetrahedron)
Avg volume per point	*Volume/NumPts* - average volume arend the localized point
ellipsoid a	Semiaxis-*a* (the longest), yielded by principal component analysis (PCA) of all localized points (wrapped)
ellipsoid b	Semiaxis-*b* (intermediate), yielded by PCA of all localized points (wrapped)
ellipsoid c	Semiaxis-*c* (the shortest), yielded by PCA of all localized points (wrapped)
a0, a1,a2	Normalized vector of sem-iaxis *ellipsoid a*
b0,b1,b2	Normalized vector of semi-axis *ellipsoid b*
c0,c1,c2	Normalized vector of sem-iaxis *ellipsoid c*
ellips aS	Semiaxis-*aS* (the longest), yielded by PCA from surface points of nucleoid/cluster (wrapped)
ellips bS	Semiaxis-*bS* (intermediate), yielded by PCA from surface points of nucleoid/cluster (wrapped)
ellips cS	Semiaxis-*cS* (the shortest), yielded by PCA from surface points of nucleoid/cluster (wrapped)
aS0, aS1, aS2	Normalized vector of semi-axis *ellips aS*
bS0, bS1, bS2	Normalized vector of semi-axis *ellips bS*
cS0, cS0, cS0	Normalized vector of semi-axis *ellips cS*
Params	Other parameters

P Ellipsoid VEquiv semiaxex aE, bE, cE	Dimensions of bounding nucleoid/cluster (wrapped) – i.e. with volume of *Volume*, and PCA semiaxes calculated from all points: *aE*=*a*/*K*, *bE*=*b*/*K*, *cE*=*c*/*K*, *K*=(*a***b***c*)^(1/3)/*Radius*
Ellipsoid VEquiv Surf semiaxes	Dimensions of bounding nucleoid/cluster (wrapped) – i.e. with volume of *Volume*, and PCA semiaxes calculated from surface points only: *aSE*=*aS*/*K*1, *bSE*=*bS*/*K*1, *cSE*=*cS*/*K*1; *K*1=(*aS***bS***cS*)^(1/3)/*Radius*

Figure S1a. **Delaunay segmentation/tesselation for a cluster –** blue surface as skeleton; channel 0, blue spheres; channel 1, red spheres.

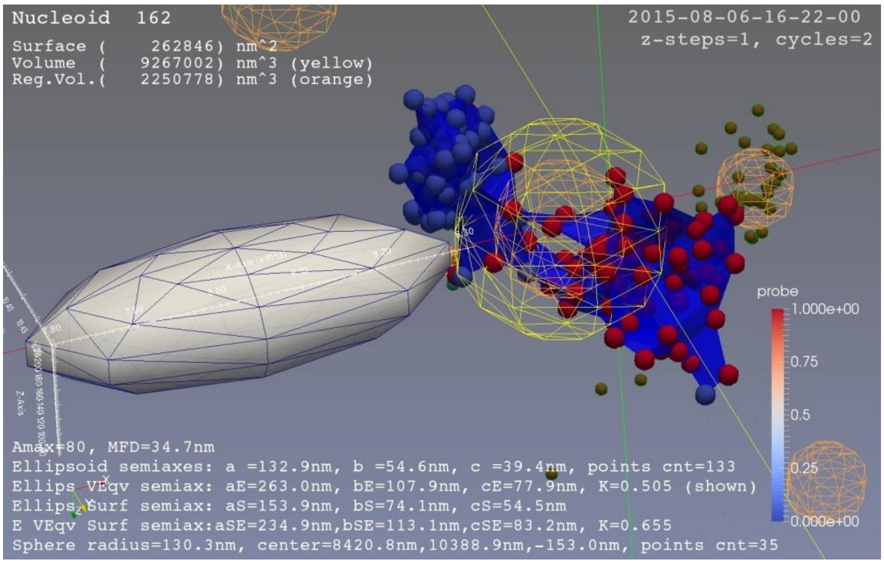

Figure S1b. **Delaunay tesselation for a cluster –** the same as above, but skinned surface in light red.

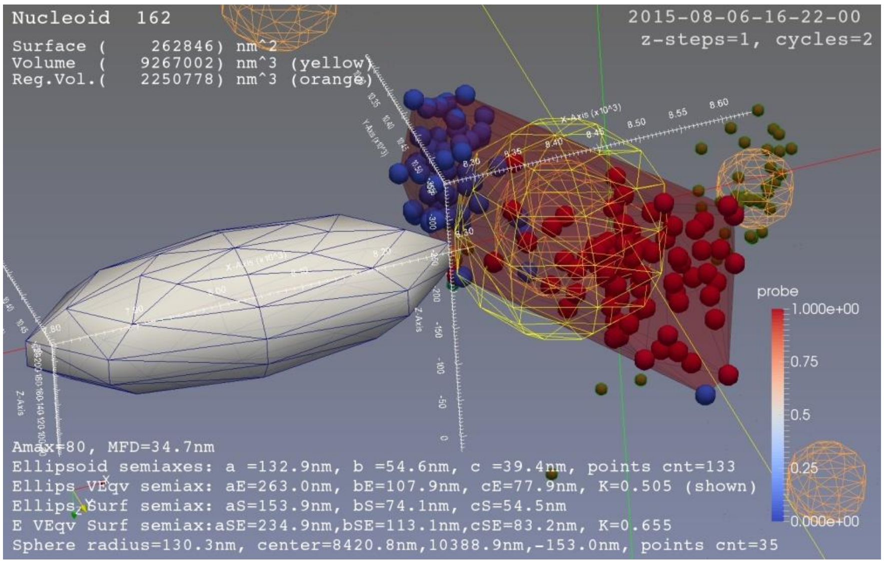

Figure S1c. **Delaunay tesselation, each channel separately –** two "sub-nucleoids", in red and blue. The farthest blue point was later filtrated from the data.

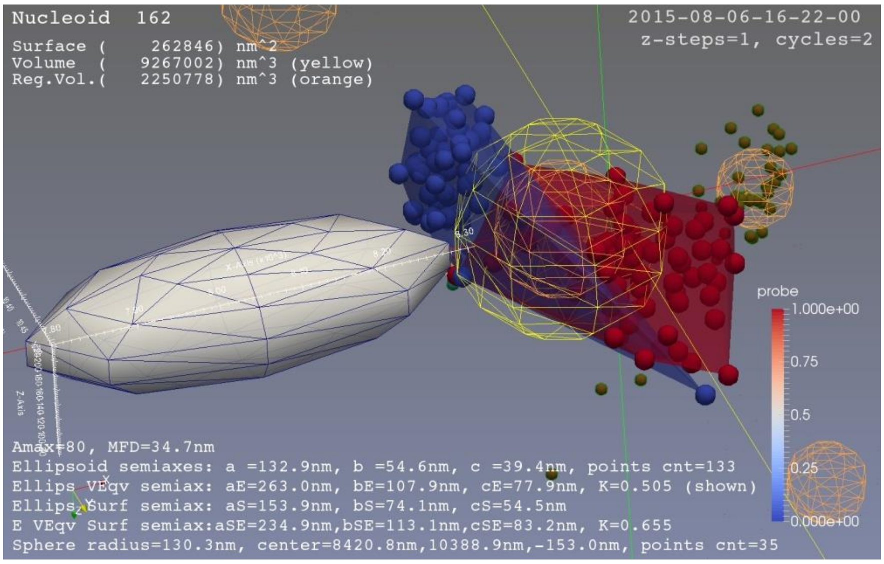

Figure S2. **Delaunay tesselation for two proximal clusters of localized points –** red and blue **–** In addition to Fig.S1C, for each channel Delaunay tesselation is performed with *Amax=80* (mean frequent diameter, MFD). Skeleton surfaces are also shown.

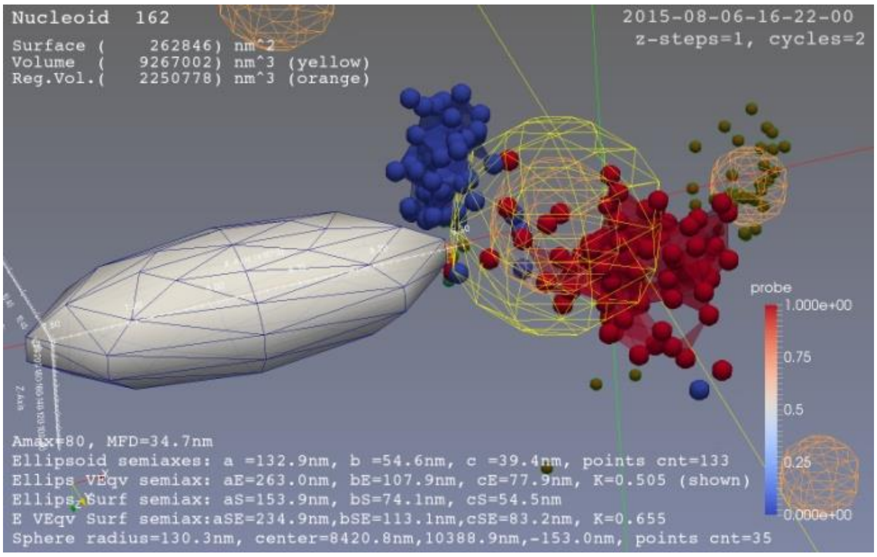

Table S2. Quantities obtained from module M3, WriteNucleotidComponents2ch.py
ALEF__nuclSurf *.csv files content

Component_Tif	Figure imaging a cluster of objects (mixed points of both channels)
Component_Pos_Tif	Figure with location of the cluster
RegionId	Identification number of the cluster
X	X-coordinate of the cluster centre of gravity
Y	Z-coordinate of the cluster centre of gravity
Z	Y-coordinate of the cluster centre of gravity
NumPts	Number of points in the cluster (nucleoid)
NumTets	Number of tetrahedrons in the cluster
Volume	Cluster volume (smoothed tetrahedrons)
Radius	Radius of sphere of equal *Volume* to the cluster volume
Surface	Cluster surface (smoothed tetrahedrons)
RegVol	Volume of cluster tetrahedrons (unsmoothed)
Volume per hexagon	*Volume/NumTets* – an average volume of hexagon in the cluster
Avg volume per point	*Volume/NumPts* – average volume around the point in the cluster
ellipsoid a	Longest semi-axis *a* – yielded from an Ellipsoid model by principal component analysis (PCA), based on all cluster points
ellipsoid b	Intermediate semi-axis *b* - Ellipsoid model by PCA based on all cluster points
ellipsoid c	Shortest semi-axis *c* - Ellipsoid model by PCA based on all cluster points
a0, a1,a2	Normalized semi-axis vector *a*
b0,b1,b2	Normalized semi-axis vector *b*
c0,c1,c2	Normalized semi-axis vector *c*
ellips aS	Longest semi-axis *aS* – yielded by PCA from points of the cluster surface (smoothed tertrahedrons)
ellips bS	Longest semi-axis *bS* – yielded by PCA from points of the cluster surface (smoothed tertrahedrons)
ellips cS	Longest semi-axis *cS* – yielded by PCA from points of the cluster surface (smoothed tertrahedrons)
aS0, aS1, aS2	Normalized vector of semi-axis *ellips aS*
bS0, bS1, bS2	Normalized vector of semi-axis *ellips bS*
cS0, cS0, cS0	Normalized vector of semi-axis *ellips cS*
X0,Y0,Z0	Coordinates of the centre of gravity in the first (nucleoid) channel – "Probe=0" inside the cluster
NumPts0	Number of points for Probe=0 in the cluster
X1,Y1,Z1	Coordinates of the centre of gravity in the second channel – "Probe=1" inside the cluster
NumPts1	Number of points for Probe=1 in the cluster; note *NumPts* = *NumPts0* + *NumPts1*
RegionCntProbe0	Number of objects (nucleoids) for Probe=0
RegionCntProbe1	Number of objects for Probe=1
RegionIdProbe	Order number of object in the cluster for Probe=0 or Probe 1
AmaxProbe0	$A_{\max}$ for Probe=0
NumPtsProbe0	Number of points for Probe=0 in the objects obtained from polyhedrons
NumTetsProbe0	Number of points for Probe=0 in the objects obtained from smoothed polyhedrons
VolumeProbe0	Volume of smoothed polyhedron for Probe=0
XR0,YR0,ZR0	Coordinates of the centre of gravity of the object *RegionIdProbe*, Probe=0
AmaxProbe1	$A_{\max}$ for Probe=1
NumTetsProbe1	Number of tetrahedrons inside the overlapping object of Probe=1
NumPtsProbe1	Number of points for Probe=1 inside the overlapp
VolumeProbe1	Volume of smoothed polyhedron for Probe=1
XR1,YR1,ZR1	Coordinates of the centre of gravity in the overlapping object with *RegionIdProbe*, Probe=1
Params	Other parameters

Note, the module writes to the file in the same directory where is the points.csv file exists The particular path is described in the Nucleoid module in the Paraview network. Thus, the file termed ALEF__nuclSurf *.csv

contains a single nucleoid (cluster) in each row. Each nucleoid (cluster) is thus defined by *RegionId*. Nucleoids or hybridization probe/antibody-localized points in a cluster from one cluster have the same *RegionId* value. Each nucleoid/cluster has ascribed a unique *RegionIdProbe*.

The created file ALEF_d *_points.csv is then a copy of "particles.csv" file with added columns. For each point, we added A_{max} and *h* of tetrahedron it belongs to. File name was created as a "mnemotechnics", for example the ALEF_d025Amax080_Points.csv portion of the file name "d025" characterizes the radius of sphere around each point, indicating the uncertainty of dSTORM imaging resolution (localization uncertainty).

Altogether, the ALEF module writes files into a subdirectory as follows:

1. _nu_*XXX_pointsYYYY.csv, where * is A_{max} or *h*; XXX max value for A_{max} or *h*; YYYY is ordinal number of cluster. For example:_nu_Amax060_points000.csv. Files include positions of all points in agiven nucleoid/cluster. Format is the same as in the file named points.csv, but with additional columns added, such as "*vtkOriginalPointIds*", "*RegionId*", "*Points:0*", "*Points:1*", "*Points:2*").
2. dXXX*YYYNuZZZ.jpg, where XXX is the diameter of a sphere around each point, * is A_{max} or *h*; XXX is the max value for A_{max} or *h*; YYYY is ordinal number of cluster. For example, the file d025Amax080Nu248.jpg includes image of cluster. Into a subdirectory ALEF\kivi module writes files *.vtp, one for each cluster.

Basic analysis:

Basic analysis is using files of type ALEF__nuclSurf *.csv, mentioned above. Data are treated by MS Access, thus yielding a file termed **ALEF_Nucleoids.xls**. In this file, each row represents characteristics of each nucleoid/cluster. **The overlapped regions are termed chunks.** Based on distance of a second Delunay sub-nucleoid-0 by A_{max}, there could be one, two and more separate regions „sub-nucleoid0". There could exist several points (having a volume) or single points (no volume). We name them chunks. Similarly for „sub-nucleoid1". **For each nucleoid, new columns are created, such as *ChunksPtsCnt0* and *ChunksPtsCnt1* , into which the number of localized points in each chunk is written. They are separated by semicolons and ordered by the higher number of points coming in front to the minimum number of points, coming as the last.**

Region Cnt Probe0	Region Cnt Probe1	Chunks Pts Cnt0	Chunks Pts Cnt1	Dist Ch0,Ch1 [nm]
19	4	4;61;56;6;4;2;2;1;1;1;1;1;1;1;1;1;1;1;1	556;2;1;1	119,6575
19	4	160;35;51;15;3;3;3;2;1;1;1;1;1;1;1;1;1;1;1	652;1;1;1	135,7905

However, in this file, the information is missing on space location of each chunk. Next, the column *DistCh0Ch1* **contains the calculated distances between centers of gravity for** Probe0 and Probe1. The file ALEF_Nucleoids.xls is also used for statistical treatment.

Software description

Software was run under Paraview version 4.3.1, 64bit.

Paraview network

Loading paraview network, ***_csvNucleoidsKmeansSurf2chSep440.pvsm*** we intend to select file with experimental data (*particles.csv* file) to read (Fig.3A). The file includes coordinates of measured points and other characteristic of data acquisition, including data channel. After network is loaded and computed, Praraview window looks like illustrated in Figure 3B.

Figure S3A Starting with a Paraview network

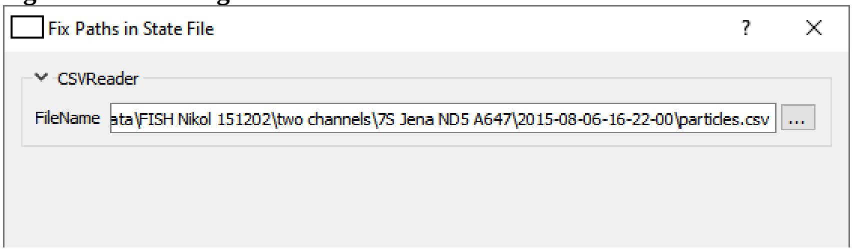

Figure S3B Resulting analysis

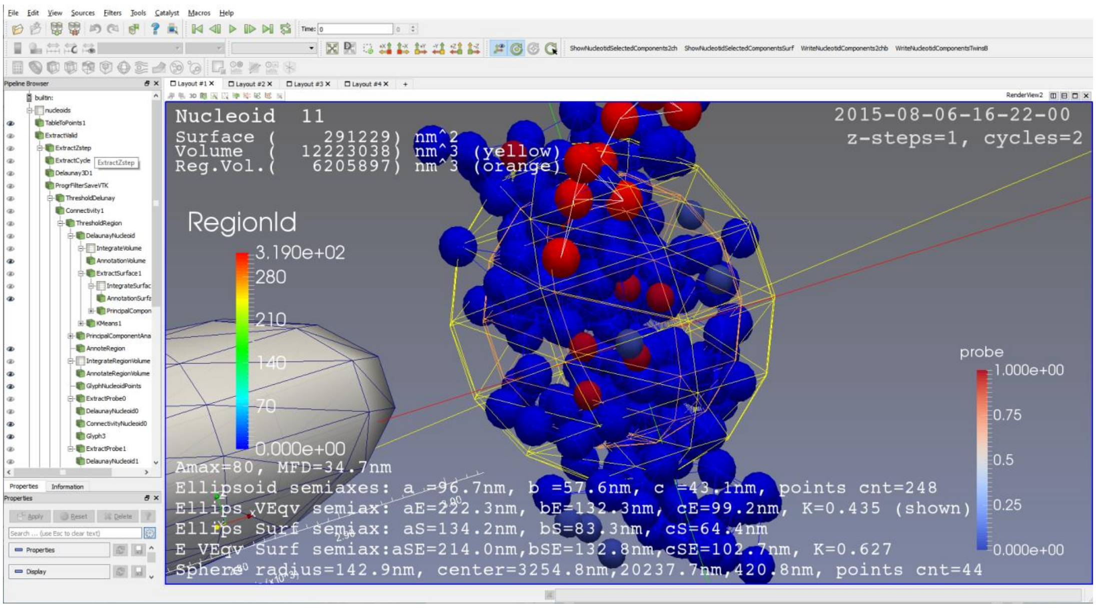

Description of Modules

Figure S4. **ThresholdDelunay module pannel**. A_{max} or h parameter used and maximum limits are set.

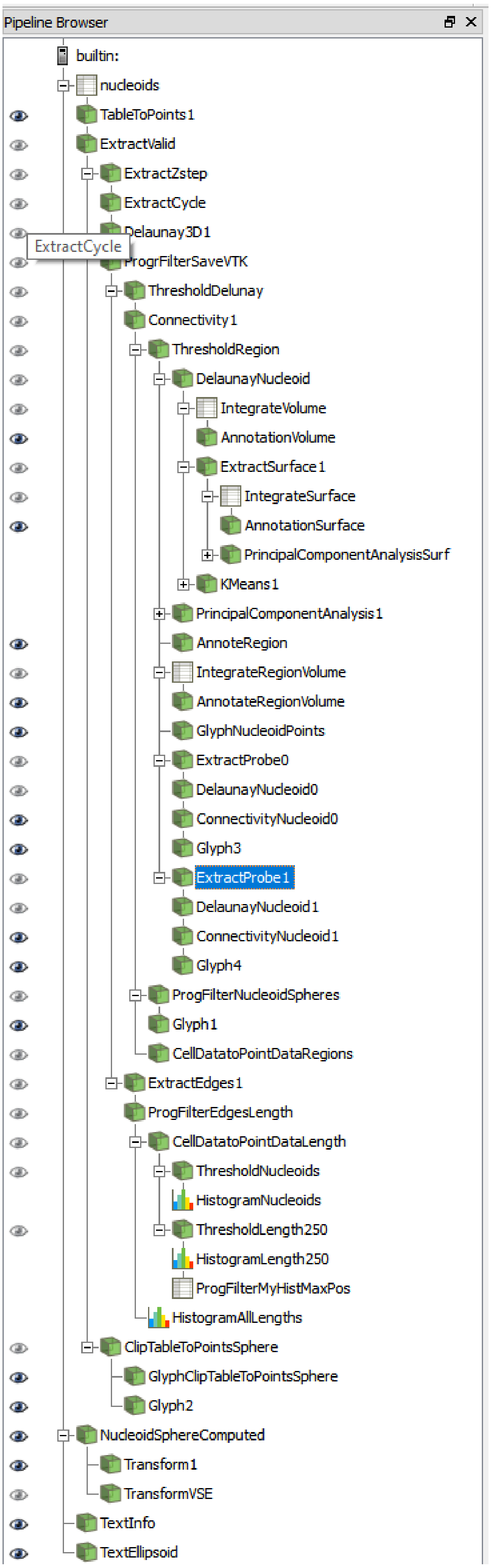

Nucleoids loads selected points.csv file. Then the ***TableToPoints1*** converts x,y,z point coordinates to the Paraview internal format.
ExtractValid is a filter which selects points from data with attribute valid=true
ExtractZstep, ***ExtractCycle*** is a filter, which allows selecting only points gathered in given Cycle or Zstep. It allows checking if there is some drift in data. But we use all points, so filter is set to include all points. We can drive this filter with macro.
The ***Delunay3D1*** computes tetrahedron mesh from points coordinates.
The ***ProgFilterSaveVTK*** is a custom filter module to compute A_{max}, h, Volume for Delunay mesh and store computed data into internal Paraview data structure (see listing.).

ThresholdDelunay includes in Delunay mesh only tetrahedrons with distance Amax or with radius h of circum sphere less then maximum value. Too far points then are not connected. As result we get separate clusters of point.

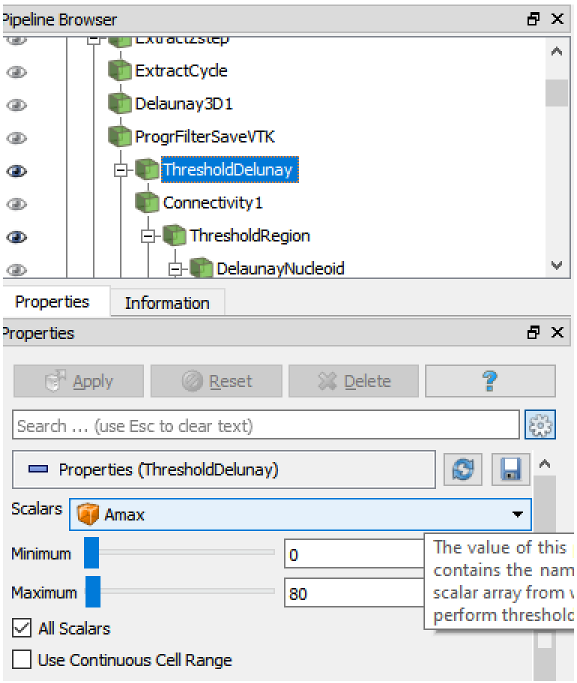

The ***Connectivity1*** divides data to connected regions (1,2,3...), each represents a single nucleoid or cluster. The ***ThresholdRegion*** filters the mesh for only the given region number (*RegionId*). The minimum and maximum must be of the same number. ***Changing***

RegionId value displays this region (nucleoid). After changing *RegionId*, the macro module ***ShowNucleotidSelectedComponents2ch.py*** is activated from the menu on a main window (Fig.S5a,b). Then the module is used (***WriteNucleotidComponents2chb.py***) to write nucleoid/cluster parameters to the csv file and nucleoid/cluster image.

Figure S5Aa. The used part of Paraview network

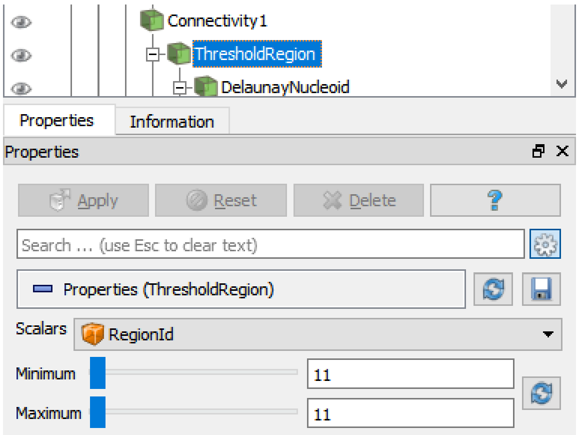

Figure S5b. Continued part of Paraview network

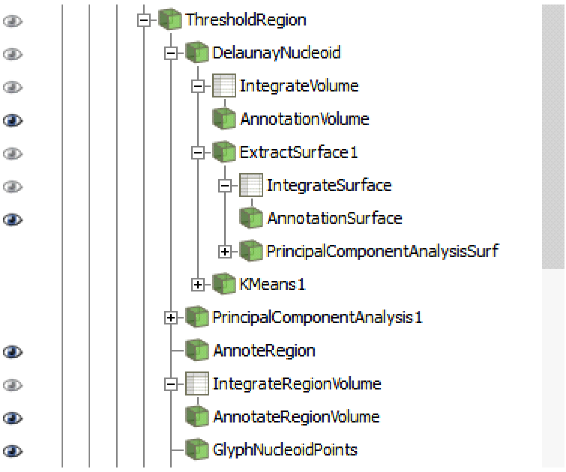

This part of paraview network (Fig.5b,c). computes again mesh from points belonging to the selected nucleoid, and computes some parameters of nucleoid plus allows to show and illustrate the nucleoide image. ***PrincipalComponentAnalysisSurf*** computes spheroid parameters, using points on a nucleoid surface only, ***PrincipalcomponentAnalysis1*** computes the same using all points in a nucleoid. Anotate* modules show text about the nucleoid and/or cluster.

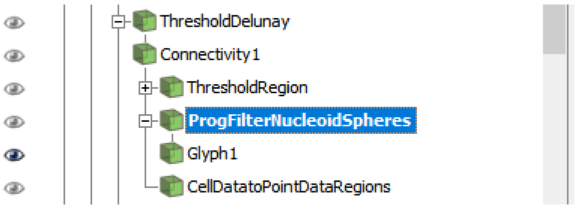

To ***Connectivity1*** module is connected to a custom module ***ProgFilterNucleoidSpheres*** (see. Module Listing). This module computes for each connected region its center and volume and appropriate sphere radius with same volume as region (e.g. nucleoide). ***Glyph1*** shows this sphere as a wire-framed sphere in orange color.

Figure S5c. Continued part of Paraview network

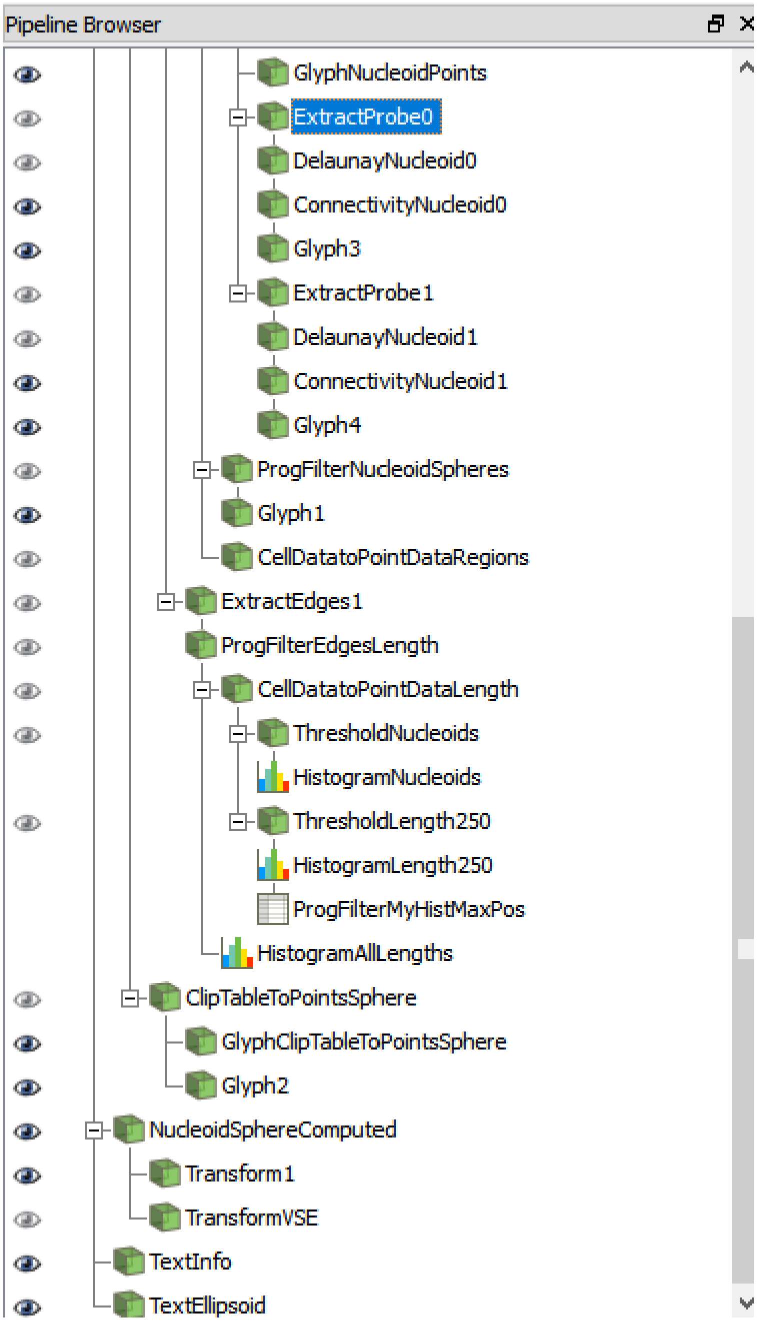

The rest of the network has several wings. Wings ***ExtractProbe0*** resp. ***ExtractProbe1*** selects points from channel 0 resp. channel 1, then works with data, as if each channel contains a separate nucleoid/cluster. ***Glyhp3*** and ***Glyph4*** display each point as a sphere in the appropriate color to distinguish each channel (red, blue).

ExtractEdges wing is then used to compute and visualize histograms of distances in nucleoid/cluster points (Fig.5d). ***ProgFilterEdgesLength*** is then the custom module to compute distances. Another module, ***ProgFilterMyHistMaxPos*** module computes position o MFD (Most Frequent Distance).

Figure S5d. Histogram visualization

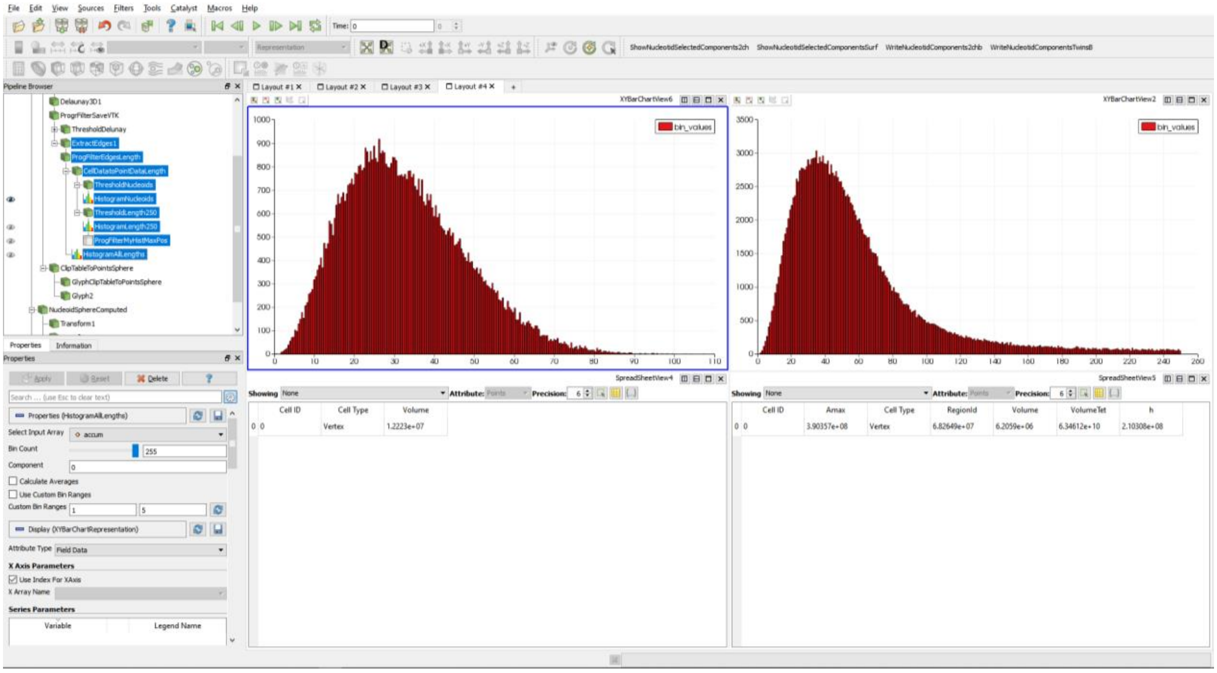

The wing ***NucleoidSphereComputed*** displays ellipsoid with the same volume as nucleoid with axes computed using ***PrincipalcomponentAnalysis1*** and ***PrincipalComponentAnalysisSurf*** , respectively. ***Transform1*** resp. ***TransformVSE*** shows the ellipsoid with that axes as white or yellow wired surface near the nucleoid. Modules **TextInfo** and **TextEllipsoid** write parameters and description to nucleoid/cluster 3D image.

Custom filters Listing

Custom filters source code is included in network yet. Here is repeated for your comfort. Modules were invented by Martin Bartos from ALEF Ltd company, Prague, Czech Republic.

ProgFilterSaveVTK list

```
# This filter computes the radius h, Amax and volume of the tetrahedra in an unstructured mesh:
 # Adapted by Johan Jansson (jjan@csc.kth.se)
 #from math import *
 #print '7.2.2014,Martin Bartos (alef@alefnet.cz)'
 pdi = self.GetInput()
 pdo = self.GetOutput()

 #copy all data to output
 pdo.ShallowCopy(pdi)

 newData = vtk.vtkDoubleArray()
 newData.SetName("h")
 newAmaxData = vtk.vtkDoubleArray()
 newAmaxData.SetName("Amax")

 #newHiData = vtk.vtkDoubleArray()
 #newHiData.SetName("hi")

 newVolData = vtk.vtkDoubleArray()
```

```
newVolData.SetName("VolumeTet")
numTets = pdi.GetNumberOfCells()
for i in range(numTets):
  cell = pdi.GetCell(i)
  #if cell.GetCellType()=VTK_TETRA:
  if cell.GetCellType()==10:
   p1 = pdi.GetPoint(cell.GetPointId(0))
   p2 = pdi.GetPoint(cell.GetPointId(1))
   p3 = pdi.GetPoint(cell.GetPointId(2))
   p4 = pdi.GetPoint(cell.GetPointId(3))
   vec12 = sum((x-y)*(x-y) for x,y in zip(p1, p2))
   vec13 = sum((x-y)*(x-y) for x,y in zip(p1, p3))
   vec14 = sum((x-y)*(x-y) for x,y in zip(p1, p4))
   vec23 = sum((x-y)*(x-y) for x,y in zip(p2, p3))
   vec24 = sum((x-y)*(x-y) for x,y in zip(p2, p4))
   vec34 = sum((x-y)*(x-y) for x,y in zip(p3, p4))
   vec = [vec12,vec13,vec14,vec23,vec24,vec34]
   A_max=vec12
   for x in vec:
    if x>A_max:
     A_max=x
   #A_max= max(y for y in vec)
   c = [0.0, 0.0, 0.0]
   h = vtk.vtkTetra.Circumsphere(p1,p2,p3,p4,c)
   h = sqrt(h)
   #hi = vtk.vtkTetra.Insphere(p1,p2,p3,p4,c)
   # VTK actually computes the square
   #vtk compute tetraheara volume
   volume = vtk.vtkTetra.ComputeVolume(p1,p2,p3,p4)
   newVolData.InsertNextValue(volume)
   newData.InsertNextValue(h)
   newAmaxData.InsertNextValue(sqrt(A_max))
   #newHiData.InsertNextValue(hi)
pdo.GetCellData().AddArray(newData)
pdo.GetCellData().AddArray(newVolData)
pdo.GetCellData().AddArray(newAmaxData)
#pdo.GetCellData().AddArray(newHiData)
```

ProgFilterEdgesLength list

```
# This filter computes points distances in each tetrahedron
# Adapted by Johan Jansson (jjan@csc.kth.se)
#from math import *
#from paraview get *
#from vtk get *

#edge = FindSource('ExtractEdges1')
#pdi = servermanager.Fetch(edge)
pdi = self.GetInput()
pdo = self.GetOutput()
#copy all data to output
pdo.ShallowCopy(pdi)

newData = vtk.vtkDoubleArray()
```

```
newData.SetName("Distance")
numLines = pdi.GetNumberOfCells()
print numLines
for i in range(numLines):
     cell = pdi.GetCell(i)
     p1 = pdi.GetPoint(cell.GetPointId(0))
     p2 = pdi.GetPoint(cell.GetPointId(1))
     #p3 = pdi.GetPoint(cell.GetPointId(2))
     #p4 = pdi.GetPoint(cell.GetPointId(3))
     vec12 = sum((x-y)*(x-y) for x,y in zip(p1, p2))
     #vec13 = sum((x-y)*(x-y) for x,y in zip(p1, p3))
     #vec14 = sum((x-y)*(x-y) for x,y in zip(p1, p4))
     #vec23 = sum((x-y)*(x-y) for x,y in zip(p2, p3))
     #vec24 = sum((x-y)*(x-y) for x,y in zip(p2, p4))
     #vec34 = sum((x-y)*(x-y) for x,y in zip(p3, p4))
     #vec = [vec12,vec13,vec14,vec23,vec24,vec34]
     distance = sqrt(vec12)
     newData.InsertNextValue(distance)

pdo.GetCellData().AddArray(newData)
```

ProgFilterMyHistMaxPos list

```
pdi = self.GetInput()
pdo = self.GetOutput()
newData = vtk.vtkDoubleArray()
newData.SetName("HistMaxPosition")
numLines = pdi.GetNumberOfRows()
hx=pdi.GetColumn(0)
hy=pdi.GetColumn(1)
Curhy=0
MaxRow=0
for i in range(numLines):
 if hy.GetValue(i) > Curhy:
  Curhy=hy.GetValue(i)
  MaxRow=i

print 'Maximum is at row {0:0.1f}, for {1:3>}
nm'.format(MaxRow,hx.GetValue(MaxRow))
newData.InsertNextValue(hx.GetValue(MaxRow))
pdo.AddColumn(newData)
```

ProgFilterNucleoidSpheres list

```
# This filter computes based on RegionId
# from Connectivity fiter
#print '7.2.2014,Martin Bartos (alef@alefnet.cz)'
# for each component
# centers and dimension
# number of points
# and creates
# polygonal mesh based on centers.
# Adapted by Johan Jansson (jjan@csc.kth.se)
import math
pdi = self.GetInput()
ido = self.GetOutput()
#copy all data to output
#ido.ShallowCopy(pdi)
numPts = pdi.GetNumberOfPoints()
newPts = vtk.vtkPoints()
#min x, y,z -bounding box
newPtsBot = vtk.vtkPoints()
newPtsTop = vtk.vtkPoints()

#Number of points in component
newPtsCnt = vtk.vtkLongArray()
#Min, Max point position in component
newPtsCnt.SetName("RegionNumPts")
#newPtsMin = vtk.vtkDoubleArray()
#newPtsMin.SetName("RegionPtsMin")
#newPtsMax = vtk.vtkDoubleArray()
#newPtsMax.SetName("RegionPtsMax")
#newPtsDelta = vtk.vtkDoubleArray()
#newPtsDelta.SetName("RegionPtsDelta")
newPtsVol = vtk.vtkDoubleArray()
newPtsVol.SetName("RegionVolume")
newPtsRad = vtk.vtkDoubleArray()
newPtsRad.SetName("RegionRadius")
newPtsDiagRad = vtk.vtkDoubleArray()
newPtsDiagRad.SetName("RegionDiagRadius")
newPtsRegion= vtk.vtkLongArray()
newPtsRegion.SetName("RegionNo")

#get RegionId data and range
pRegion=pdi.GetPointData().GetScalars()
pRegiRange=pRegion.GetRange()
pRegiCnt=int(pRegiRange[1]+1)

#get cell data to allow sum volume
numCells = pdi.GetNumberOfCells()
cCellData=pdi.GetCellData().GetScalars()
#print(dir(cCellData))
#initialize center positions array
#initialize point incomponent count array
for i in range(0,pRegiCnt):
  newPts.InsertNextPoint(0,0,0)
  newPtsBot.InsertNextPoint(100000,100000,100000)
  newPtsTop.InsertNextPoint(-100000,-100000,-100000)
```

```
  newPtsCnt.InsertNextValue(0)
  #newPtsMin.InsertNextValue(0)
  #newPtsMax.InsertNextValue(0)
  newPtsVol.InsertNextValue(0)
  newPtsRegion.InsertNextValue(i)

#compute sum, min, max of of Input points positions
#newPtsMin.SetValue(0,pdi.GetPoint(0)[0])
#newPtsMax.SetValue(0,pdi.GetPoint(0)[0])

for i in range(0,numPts):
 j=int(pRegion.GetValue(i))
 a=newPts.GetPoint(j)
 b=pdi.GetPoint(i)
 c= a[0]+b[0],a[1]+b[1],a[2]+b[2]
 newPts.SetPoint(j,c)
 a=newPtsCnt.GetValue(j)
 newPtsCnt.SetValue(j,a+1)
 #compute x min, max
 #d=newPtsMin.GetValue(j)
 #if d>b[0] or d==0 :
 # newPtsMin.SetValue(j,b[0])
 #d=newPtsMax.GetValue(j)
 #if d<b[0] or d==0 :
 # newPtsMax.SetValue(j,b[0])

 e=newPtsBot.GetPoint(j)
 Bot=min((b,e))
 newPtsBot.SetPoint(j,Bot)

 e=newPtsTop.GetPoint(j)
 Top=max((b,e))
 newPtsTop.SetPoint(j,Top)

# compute volume of component
cRegion=pdi.GetCellData().GetArray('RegionId')
#print 'cRegion',cregion

#volum sum Works Well only for tetrahedra
for i in range(0,numCells):
 j= cRegion.GetValue(i)
 #if pdi.GetCell(i).GetCellType()=10:
 V= newPtsVol.GetValue(j)
 #v1=pdi.GetCellData().GetArray('Volume').GetValue(i)
 cell = pdi.GetCell(i)
 #if cell.GetCellType()==VTK_TETRA:
 if cell.GetCellType()==10:
  newPtsVol.SetValue(j,V+pdi.GetCellData().GetArray('VolumeTet').GetValue(i))


#Compute center, delta and average and radius based on volume
for i in range(0,pRegiCnt):
 a=newPts.GetPoint(i)
 b=newPtsCnt.GetValue(i)
 c= a[0]/b,a[1]/b,a[2]/b
 newPts.SetPoint(i,c)
```

```
 #newPtsDelta.InsertNextValue(newPtsMax.GetValue(i)-newPtsMin.GetValue(i))
 newPtsRad.InsertNextValue((3./4. * newPtsVol.GetValue(i)/pi)**(1./3.))
 #Bounding Box diagonal
 #newPtsDiagRad.InsertNextValue((sum((x-y)**2 for x,y in
zip(newPtsTop.GetValue(i),newPtsBot.GetValue(i))))**0.5)
 Top=newPtsTop.GetPoint(i)
 Bot=newPtsBot.GetPoint(i)
 newPtsDiagRad.InsertNextValue((((Top[0]-Bot[0])**2+(Top[1]-Bot[1])**2+(Top[2]-
Bot[2])**2)**0.5)/2)
 #print newPtsDiagRad.GetValue(i)

ido.SetPoints(newPts)
ido.GetPointData().AddArray(newPtsCnt)
#ido.GetPointData().AddArray(newPtsMin)
#ido.GetPointData().AddArray(newPtsMax)
ido.GetPointData().AddArray(newPtsVol)
ido.GetPointData().AddArray(newPtsRad)
ido.GetPointData().AddArray(newPtsDiagRad)
ido.GetPointData().AddArray(newPtsRegion)


#Create output
#ido.SetDimensions(pRegiCnt,3,3)
#ido.SetOrigin(-1,-1,-1)
#ido.SetSpacing(.1,.1,.1)
#ido.SetWholeExtent(0,numPts,0,1,0,1)
#ido.AllocateScalars()
#debug
print 'NumPoints',(numPts)
print 'Num of Regions ',(pRegiCnt)
print '*'

print('Finished')
```

Macros

You can add macro to Paraview using macro -> Add new macro form Paraview menu. Macro is then added to Paraview menu.

Macros depend on names of modules in active network. Macros here are designed to work with network ***_csvNucleoidsKmeansSurf2chSep440.pvsm***:

File	Description
ShowNucleotidSelectedComponents2ch.py	Macro to Show selected nucleoid
WriteNucleotidComponents2ch.py	Macro to Write csv and jpg for each nucleoid in experimental data file

ShowNucleotidSelectedComponents2ch.py

When you select nucleoide changing parameter *RegionId* in ***ThresholdRegion*** module panel (do not forget to press 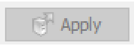

), the required nucleoid is not visible, because is usually out of view. Press 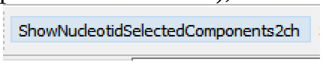

to move view to selected nucleoid and to show nucleoid calculated parameters.

Automation

Manual data analysis i time consuming. We prepared the script

AutomateNetwork FISH Nikol 151202 Double Single Color.py.

Run Paraview and load network ***_csvNucleoidsKmeansSurf2chSep440.pvsm.*** From a menu select Tools-> Python Shell. Here choose RunScript to initiate the skript ***AutomateNetwork FISH Nikol 151202 Double Single Color.py.***

Figure S6. Automation of the procedure

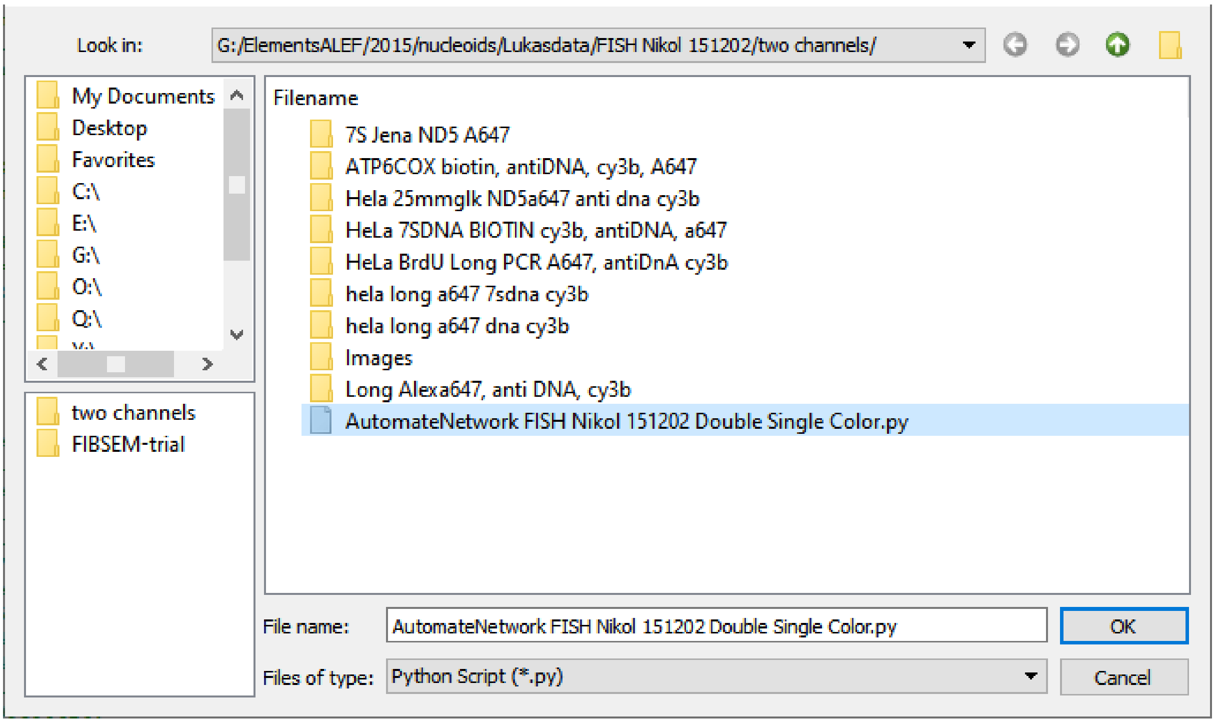

The filed defines function *AnalyseComponents(csvFileName)*, which reads the chosen csv file for actual Paraview network. Alwaus, the nework must be located is under the umbrella of ***_csvNucleoidsKmeansSurf2chSep440.pvsm***) . This will call a macro termed as ***WriteNucleotidComonents2chb***, which will calculate the requested items and will store the data to the csv and jpg files.
The main programme then only calls this function for each exectude data file. For example:

AnalyseComponents('//A7xp/elementsalef/2015/nucleoids/Lukasdata/FISH Nikol 151202/two channels/hela long a647 7sdna cy3b/2015-08-13-16-55-55/particles.csv')
AnalyseComponents('//A7xp/elementsalef/2015/nucleoids/Lukasdata/FISH Nikol 151202/two channels/hela long a647 dna cy3b/2015-08-11-14-31-52/particles.csv')
AnalyseComponents('//A7xp/elementsalef/2015/nucleoids/Lukasdata/FISH Nikol 151202/two channels/hela long a647 dna cy3b/2015-08-11-14-37-28/particles.csv')

The execution, however, lasts typically tens of hours.¨When finished, all results are ready and finished in csv files and nucleoid /cluster images in image files.

Release Notes

Due to the error in the Paraview software spreadsheet, a view of data does not remember its source data. Due this error, the appropriate views are not initialized. Hence, below we add screenshots, to see what data should be displayed (Fig.S7). To correct it do as follows:

1. Activate spreadsheet clinking on it by mouse.
2. Check in network window, which module should be displayed (the eye left from module is bold)
3. Click on this eye once to stop the diplay module and then again to display it. The view is then corrected}. The header of the selected spreadsheet should be the same as is in the screenshot. Happily, this error has no influence on data witted by automation.

Figure S7a. Screenshot of layout 2

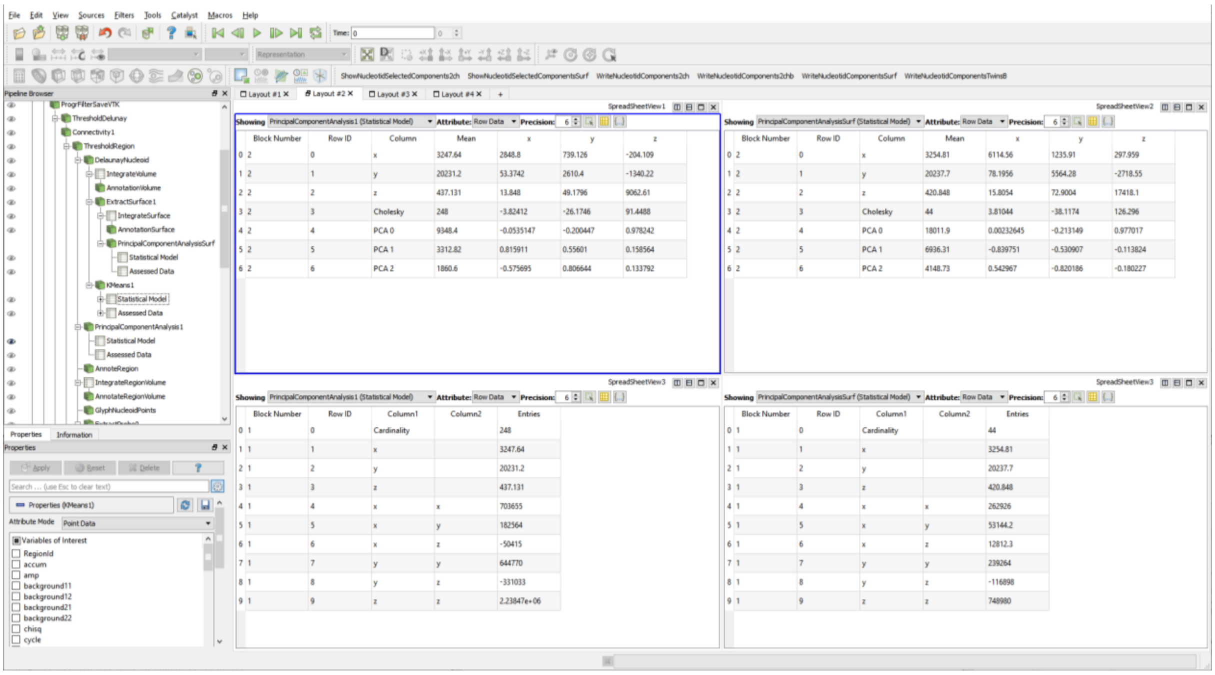

Figure S7b. Screenshot of layout 3

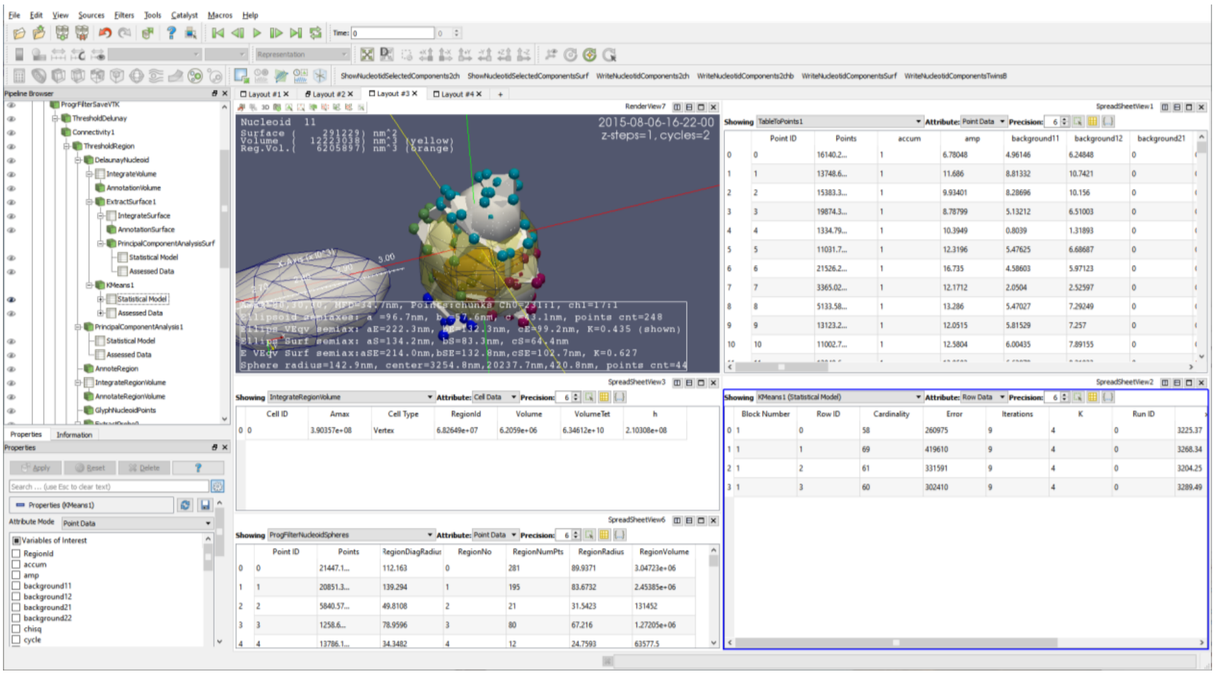

Figure S7c. Screenshot of layout 4

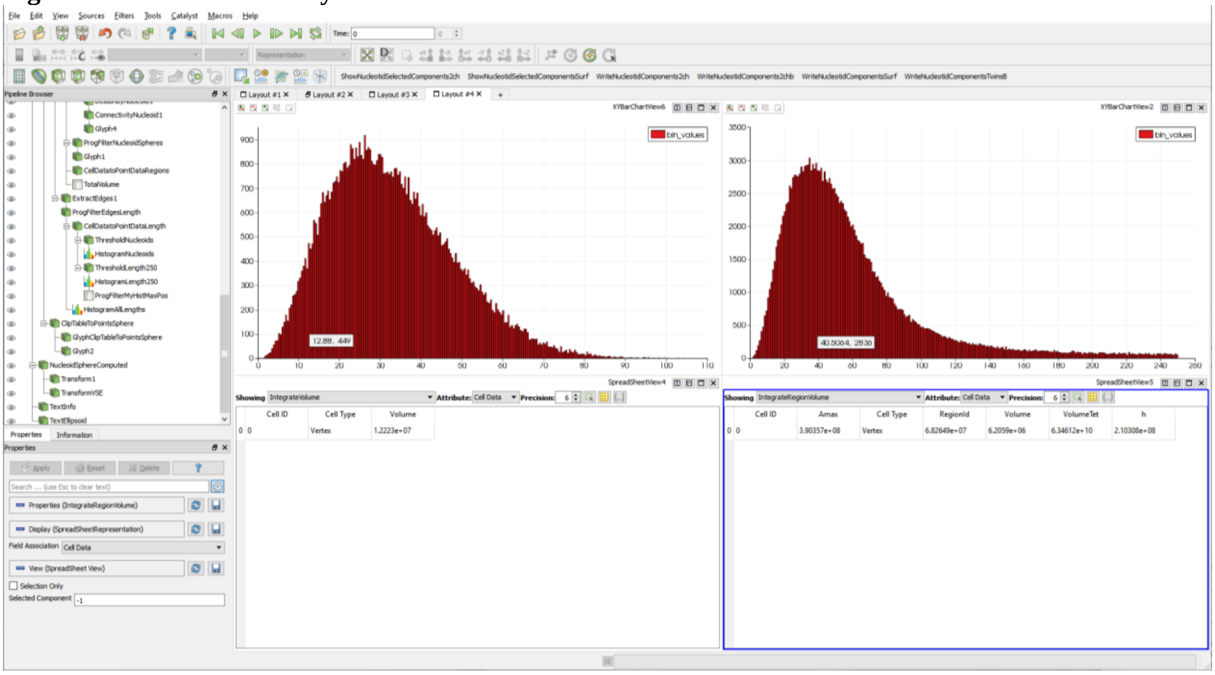

PART II AUXILIARY DATA AND VISUALIZATION Of DELAUNAY TESSELATION

PERFORMANCE OF VUTARA SRX INSTRUMENT

As described in Methods, imaging was performed on a biplane-mode fluorescence photoactivated localization microscopy (FPALM) instrument [38–40,48], a prototype of Vutara (formerly Vutara, Salt Lake City, UT, USA; now Bruker Nanosurface, Middleton, WI, USA), equipped with lasers (Coherent, Santa Clara, CA, USA) emitting at 405 nm (Obis 405), 488 nm (Sapphire 488-200), 561 nm (Sapphire 561-200) and 641 nm (Cube 640-100).

Besides decorrelation analysis [53] (cf. Fig.2 legend), another example of real experimental resolution in the xy-plane was estimated using expression of Eos-conjugated truncated FIS1 protein, which localizes to the outer mitochondrial membrane (see Ref. [39]). Thus the typical image of hollow tubules of mt network is illustrated (Fig.S8a) with the achieved resolution of 35 nm in the xy-plane, estimated as a full-width at half-maximum (FWHM) of a cylinder edge within the particle position histogram of crossection (Fig.S8b).

Figure S8 – a) Localized Eos-FIS1; b) Estimation of 35 nm resolution as derived from the width of the steep edge of a histogram, counting the localized positions.

We illustrate this FPALM imaging of a cylindrical tubule having 180 nm in diameter to show that a cylindrical surface of membrane is visualized with a spatial resolution of 35 nm. Note that mt tubule is not a perfect cylinder, so that the edge can be widened due to uneven cylindrical surface.

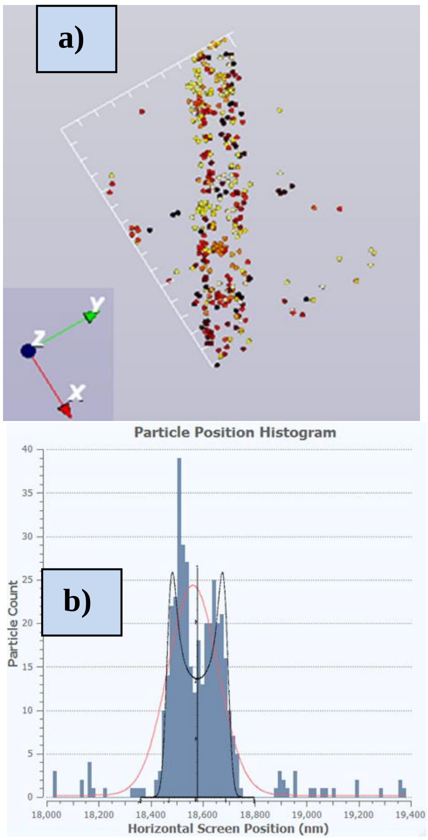

3D IMAGE PROCESSING BY DELAUNAY TESSELLATION

The Delaunay tessellation procedure is illustrated in Fig.S9 by the three steps when using an increasing base A_{max} of tetrahedrons (green). In each of steps a) to c) resulting spheroid models are drawn for a transient candidate nucleoid (light blue). Example is illustrated with 3D dSTORM raw data (white points), obtained using anti-TFAM antibodies with the secondary antibodies conjugated with Alexa Fluor 647.

Figure S9 –Illustration of three-step tesselation

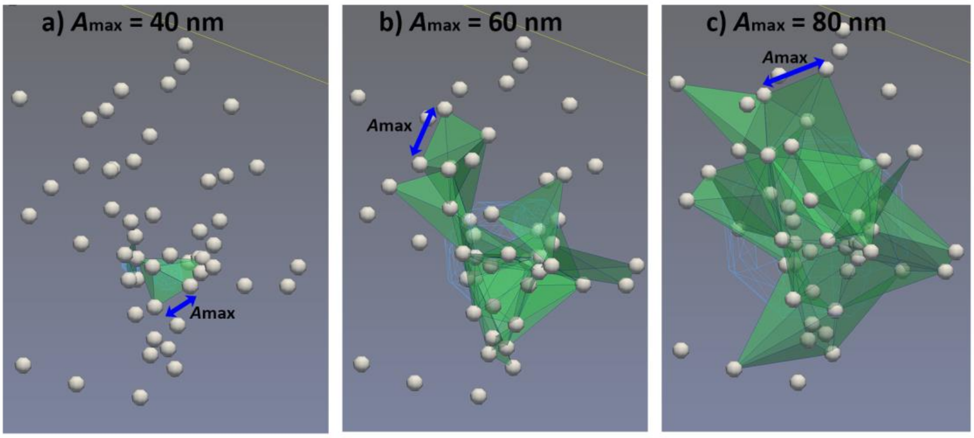

3D IMAGE PROCESSING - UNIFIED VS. CALCULATED ACCURACY OF LOCALIZATION

Vutara SRX software allows to plot the localized fluorophores either with a unified accuracy of localization or with calculated accuracy of localization for each localized fluorophore. Comparison of both plots is illustrated in Fig.S10. For the purpose of the above described segmentation based on Delaunay algorithm, however, both types of localization data give nearly identical segmentation for objects such as nucleoids when constructed with 80 to 500 localizations. This is also similar for tessellation of both channels together. With accuracy plots one could even recognize more “chunks: of mtFISH localization, so with our approach, rather a minimum limit is estimated.

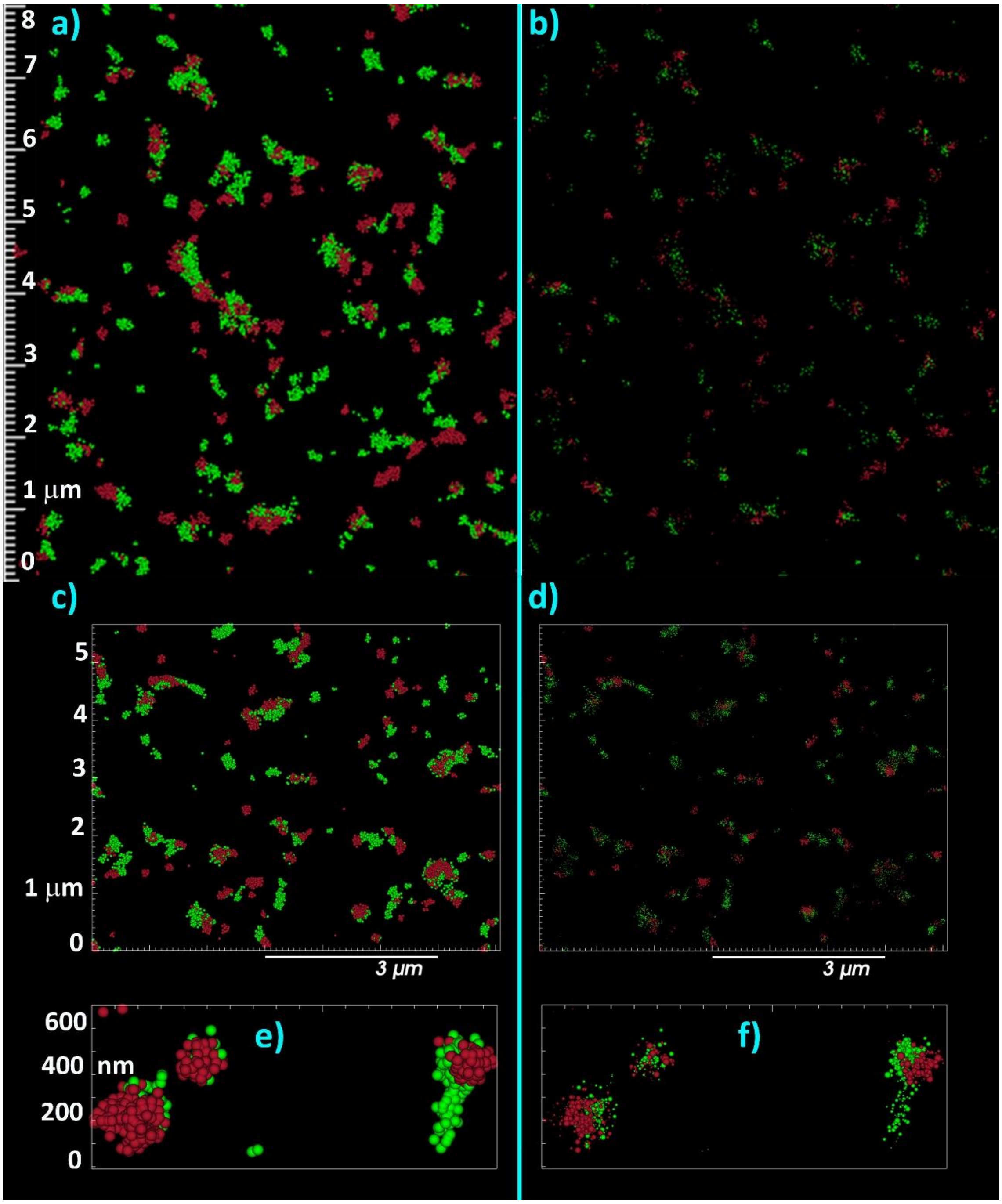

Figure S10 – a),c),e) unified accuracy; b),d),f) calculated accuracy for each localization for 3D dSTORM data of mtFISH with the DLOOP-biotin Cy3B (red) *vs*. antiDNA-A647 (green) protocol.
